# Next-Generation Desalination Membranes Empowered by Novel Materials: Where Are We Now?

**DOI:** 10.1007/s40820-024-01606-y

**Published:** 2024-12-20

**Authors:** Siqi Wu, Lu Elfa Peng, Zhe Yang, Pulak Sarkar, Mihail Barboiu, Chuyang Y. Tang, Anthony G. Fane

**Affiliations:** 1 Department of Civil Engineering, The University of Hong Kong, Pokfulam, Hong Kong SAR, People’s Republic of China; 2 Institut Européen des Membrane, University of Montpellier, ENSCM, CNRS UMR5635, Place Eugène Bataillon, CC 047, 34095 Montpellier, France; 3 UNESCO Centre for Membrane Science and Technology, School of Chemical Engineering, The University of New South Wales (UNSW), Sydney, NSW 2052 Australia

**Keywords:** Novel materials, Desalination membranes, Reverse osmosis, Evaluation framework, Separation performance

## Abstract

Membrane desalination is an economical and energy-efficient method to meet the current worldwide water scarcity. However, state-of-the-art reverse osmosis membranes are gradually being replaced by novel membrane materials as a result of ongoing technological advancements. These novel materials possess intrinsic pore structures or can be assembled to form lamellar membrane channels for selective transport of water or solutes (e.g., NaCl). Still, in real applications, the results fall below the theoretical predictions, and a few properties, including large-scale fabrication, mechanical strength, and chemical stability, also have an impact on the overall effectiveness of those materials. In view of this, we develop a new evaluation framework in the form of radar charts with five dimensions (i.e., water permeance, water/NaCl selectivity, membrane cost, scale of development, and stability) to assess the advantages, disadvantages, and potential of state-of-the-art and newly developed desalination membranes. In this framework, the reported thin film nanocomposite membranes and membranes developed from novel materials were compared with the state-of-the-art thin film composite membranes. This review will demonstrate the current advancements in novel membrane materials and bridge the gap between different desalination membranes. In this review, we also point out the prospects and challenges of next-generation membranes for desalination applications. We believe that this comprehensive framework may be used as a future reference for designing next-generation desalination membranes and will encourage further research and development in the field of membrane technology, leading to new insights and advancements.

**Supplementary Information:**

The online version contains supplementary material available at 10.1007/s40820-024-01606-y.

## Highlights

- The theoretical separation performance and practical separation performance of various membranes were collected and compared.
- An up-to-date holistic and systematic evaluation of membranes from five dimensions (i.e., water permeance, water/NaCl selectivity, membrane cost, scale of development, and stability) is provided and visualized by radar charts.
- The critical deficiencies revealed in the review are important in guiding the development of next-generation reverse osmosis membranes.

**Supplementary Information:**

The online version contains supplementary material available at 10.1007/s40820-024-01606-y.

## Graphical abstract

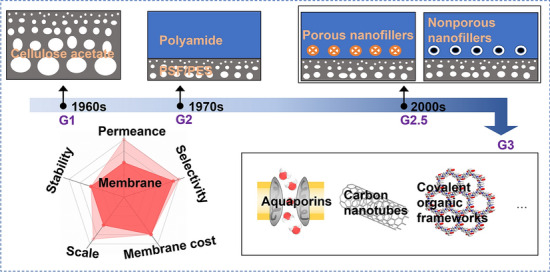

## Introduction

Water scarcity is an unavoidable challenge due to the global population explosion, industrialization, and climate change [1, 2]. To mitigate this crisis, desalination and water reuse by reverse osmosis (RO) technology [3, 4] have been increasingly adopted. RO technology relies on membrane materials that can selectively remove small solutes, even monovalent salts, from aqueous solutions [5]. Currently, the thin-film composite (TFC) membranes being used in RO processes are predominately composed of polyamide-based materials. These polyamide membranes are limited by permeance selectivity trade-off [6–9], chlorine attack [10–12], and membrane fouling [12–14], which can be attributed to the inherent material properties of polyamide chemistry. Therefore, alternative advanced membrane materials are highly desired to further develop RO membranes.

A wide variety of novel materials have been explored for making high-performance RO membranes. For example, the naturally occurring aquaporins (AQPs), when incorporated into amphiphilic triblock-polymer vesicles, exhibited a water permeance of 167 μm s^−1^ bar^−1^, which is two orders of magnitude higher than the water permeance of the current polyamide-based TFC membranes [15]. Inspired by AQPs, artificial water channels (AWCs) constructed by simpler synthetic compounds when embedded in the polyamide layer demonstrated their effectiveness in improving separation performance and fouling resistance of membranes [16, 17]. Many other materials, such as carbon nanotubes (CNTs) [18, 19], nanoporous graphene [20], and stacked two-dimensional (2D) materials [21, 22], also have good implications in separation performance, chemical stability, and/or fouling resistance.

Although many research papers have reported the exciting performance of novel materials, they may not accurately reflect the overall separation performance in the RO process, and some critical characteristics of the membrane materials (e.g., cost, scale, stability) were overlooked. For instance, the results are not comparable in some situations because these materials were tested in a concentration-driven process [23] or thermal-driven process [24] instead of the pressure-driven RO process. In other cases, simulation has provided exciting results of materials, but some traits of these materials which can greatly influence the performance were ignored, such as the flexibility of metal–organic frameworks (MOFs) [25–27]. On the other hand, certain membrane fabrication processes, like the preparation of vertically aligned CNT (VA-CNT) membranes, which involve chemical vapor deposition (CVD) and complicated fabrication procedures [28], are difficult to scale up for industrial uses. Thus, we require a standard framework to assess various membrane materials and contrast them in all relevant dimensions.

There exist several review papers [8, 29, 30] that provide qualitative narrations on membrane materials, but a more quantitative framework is still needed. Pendergast et al. [31] provided a useful semi-quantitative assessment of water treatment membranes based on performance enhancement and commercial readiness, but the work was done one decade ago, and membrane technology has advanced much since then. In this paper, we will first briefly introduce the status of novel materials for RO membranes. Then, we will provide our critical evaluations of these materials based on their separation performance and further benchmark them from all-around dimensions. Finally, we will provide suggestions for future RO membrane development. In short, we intend to provide an up-to-date holistic and systematic evaluation of emerging membrane materials. The critical deficiencies of these membrane materials revealed in this review call for more attention from future research, which will be of great importance in guiding the development of next-generation high-performance RO membranes.

## Novel Materials with Potential for RO Membranes

**Fig. 1:**
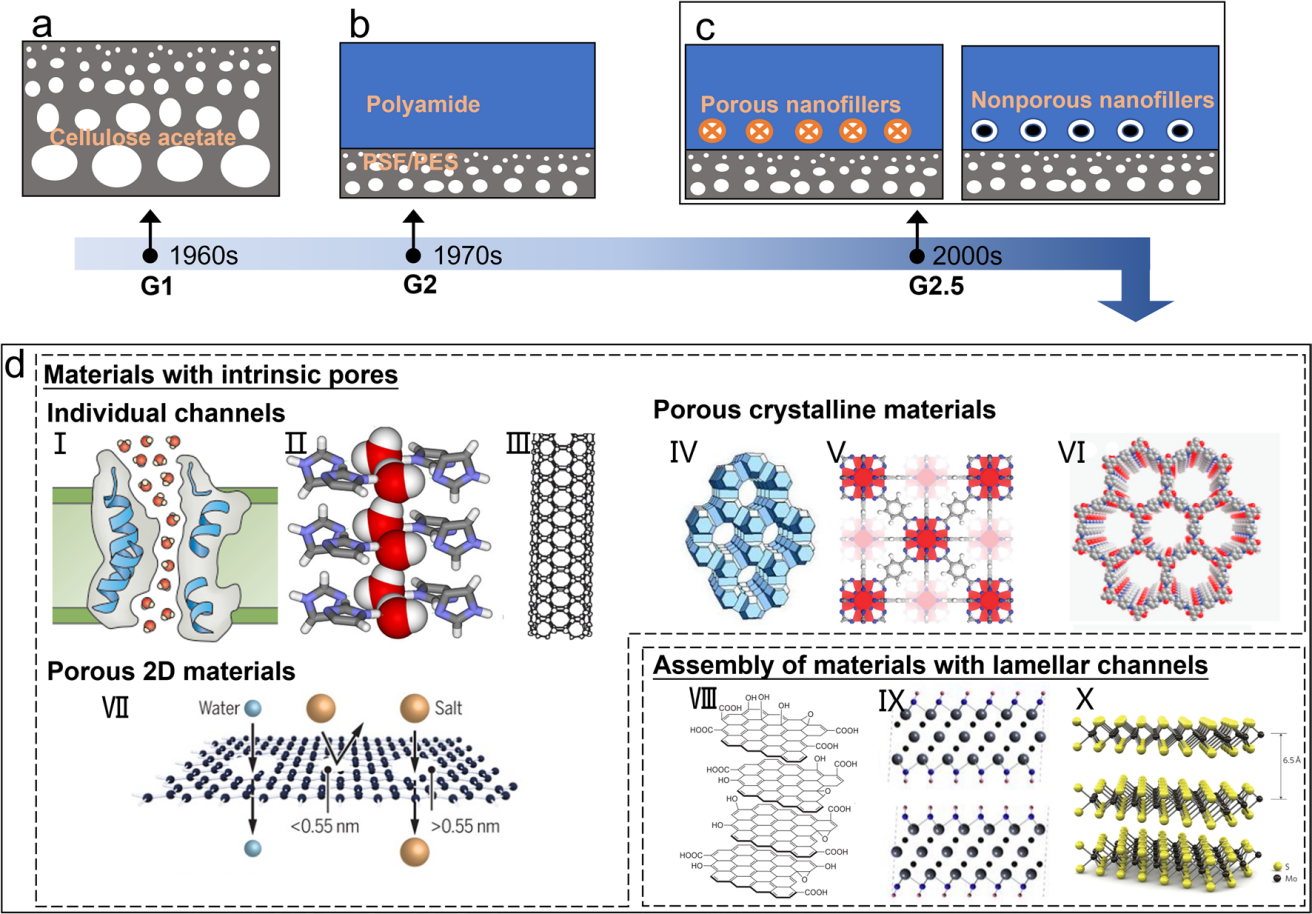
Development of RO membrane materials. Schematic illustration of **a** cellulose acetate membrane, **b** polyamide-based TFC membrane, and **c** TFN membranes with porous/non-porous nanofillers. **d** Novel materials with potential for RO membranes: (I) AQP subunit; (II) I-quartet water channels that selectively accommodate water-wires, adapted with permission from Ref. [51], copyright © 2013, American Chemical Society; (III) CNT; (IV) zeolite ZSM-5 crystal structure [52]; (V) UiO-66 (Zr, O, C, and H atoms are in red, blue, gray, and white respectively), reproduced with permission from ref. [53], copyright © 2008, American Chemical Society; (VI) COF TpPa-1 (C, O, and H atoms are in grey, red, and blue, respectively), reproduced with permission from ref. [54], copyright © 2012, American Chemical Society; (VII) nanoporous graphene, reproduced with permission from Ref. [55], copyright © 2019, The American Association for the Advancement of Science; (VIII) stacked GO nanosheets, reproduced with permission from Ref. [56], copyright © 2020 Elsevier Inc.; (IX) stacked MXenes nanosheets (Ti, C, O, and H atoms are in grey, black, blue, and pink, respectively), reproduced with permission from Ref. [57], copyright © 2011, WILEY–VCH Verlag GmbH & Co. KGaA, Weinheim; (X) stacked MoS_2_ nanosheets (Mo and S atoms are in blue and yellow respectively), reproduced with permission from Ref. [58], copyright © 2022, Wiley–VCH GmbH

Historically, the first-generation (G1) of practically selective cellulose acetate RO membranes–with an asymmetric structure (Fig. 1a)–was introduced in the 1960s [32]. Despite achieving NaCl rejection of up to 99%, cellulose acetate membranes generally have low water permeance, narrow operation range (e.g., pH within 4–6), and poor resistance to biodegradation [31, 33]. Due to these critical limitations, the G1 cellulose acetate membranes were soon replaced by TFC polyamide membranes [34], the second-generation (G2) RO membranes (Fig. 1b). TFC polyamide membranes produced by interfacial polymerization (IP) represent the state-of-the-art desalination membranes, with modern commercially available TFC RO membranes featuring water permeance of ~ or > 1 L m^−2^ h^−1^ bar^−1^, NaCl rejection of > 99%, and a typical operational pH range of 3–10 (with wider pH ranges possible for tailor-designed TFC membranes). Nevertheless, the polyamide-based TFC RO membranes are still constrained by a strong permeance-selectivity trade-off [6–9], generally showing compromised selectivity for membranes with greater water permeance. Key factors in the trade-off behavior are the structure and properties of the polyamide selective layer, such as pore size distribution and crosslinking density. For example, increasing the crosslinking degree reduces the effective pore size, which tends to improve membrane rejection and selectivity at the expense of reduced water permeance. The polyamide active layer, an irregularly crosslinked amides network, typically contains unevenly distributed pores, including both smaller network pores and larger aggregate pores [35]. This mal-distribution of pore size for polyamide, in contrast to the well-defined and uniformly-distributed pores for many emerging porous materials such as AQPs and MOFs (Table 1), tends to adversely affect membrane rejection and ultimately limit the selectivity of polyamide G2 membranes. In addition, these G2 membranes are prone to chlorine attack [10–12] and membrane fouling [12–14], causing decreased performance and lifespan of the membranes. More specifically, polyamide membranes can degrade when exposed to chlorine, as chlorine can break down the amide groups in the polyamide structure, leading to reduced effectiveness in removing salt and other impurities.

**Table 1:** Comparison of various porous materials with potential for RO membranes

Type	Materials	Size of pores/channels	Water transport and selectivity ^a^	Remarks
Individual channels	AQPs	AQPZ [79]: 2.8 Å	AQPZ [80]: ~ 3 × 10^9^ water molecules per subunit per second, blocking most solutes, including small molecules such as glycerol, urea, and sorbitol (stopped-flow light-scattering measurements)	Other AQPs have also been reported. For example, AQP1 [59] shows similar water transport and selectivity with AQPZ
	AWCs	PAPs (peptide-appended pillar[5]arenes) [69]: ~ 5 Å I-quartets [67]: 2.6 Å AWC1 (fluorofoldamer-based AWCs) [71]: 5.2 Å	PAPs [69]: 3.5 × 10^8^ water molecules per channel per second, transport water and cations I-quartets [67]: ~ 10^6^ water molecules per channel per second, transport water and protons AWC1 [71]: 1.4 × 10^10^ water molecules per channel per second, block salts and protons	Water transport and selectivity are based on stopped-flow light-scattering measurements
	CNTs	Diameter typically in the range of 0.4 nm [81, 82] to ~ 1.5 nm; large diameter resulting in poorer salt rejection	Narrow 0.8-nm-diameter CNT porins (effective pore diameter: 0.68 nm) [74]: 2.3 × 10^10^ water molecules per channel per second, transport water and cations (stopped-flow light-scattering measurements)	The antibacterial property of CNTs [75] is sometimes used for the preparation of anti-biofouling membranes
Porous crystalline structures	Zeolites	ZSM-5 [83]: 5.4 Å × 5.6 Å (the size of straight channels) ZK-4 [84]: 4.2 Å NaA [85]: 4 Å	ZK-4 [84]: 100% NaCl rejection (MD simulation) TFN membrane with NaA zeolite as nanofillers achieved nearly doubled water permeance while keeping the NaCl rejection compared to the original TFC membrane [38]	Rejection of ZK-4 is based on MD simulation
	MOFs	UiO-66 [53]: 6 Å ZIF-8 [27]: 11.6 Å	UiO-66 [86]: 51.05 L m^−2^ h^−1^ bar^−1^, 100% NaCl rejection (MD simulation) ZIF-8 [27]: ~ 170 L m^−2^ h^−1^ bar^−1^, 100% NaCl rejection (MD simulation)	Separation performance is based on MD simulations Most kinds of MOFs are not stable in water. However, MOFs made by high valence metal ions or imidazolate-based organic linkers could be stable in water
	COFs	LZU1 [87]: 1.8 nm TpPa-1 [54]: 1.83 nm DhaTab [88]: 3.7 nm CTF-1-CH_3_ [89]: 5.98 Å TpPa-1 (stacked in the offset eclipsed fashion) [90]: 0.89 nm 3D-OH-COF [91]: 7 × 18 Å^2^	CTF-1-CH_3_ [89]: 1025 ± 133 L m^−2^ h^−1^ bar^−1^, 100% NaCl rejection (MD simulation) TpPa-1 (stacked in the offset eclipsed fashion) [90]: 1118 L m^−2^ h^−1^ bar^−1^, 100% MgCl_2_ rejection (MD simulation) 3D-OH-COF [91]: 1727 L m^−2^ h^−1^ bar^−1^, 100% NaCl rejection (MD simulation)	Separation performance is based on MD simulations Compared to MOFs, COFs have larger pore sizes (which would result in poor ion rejection). To reduce the pore size of COFs, the addition of functional groups (e.g., CTF-1-CH_3_, 3D-OH-COF) or special stacking fashion has been investigated
Porous 2D materials	Nanoporous graphene	Typical range of pore size: from a few angstroms to a few nanometers [92–97]	Simulation of a 16.3 Å^2^ hydroxylated pore [98]: 2750 L m^−2^ h^−1^ bar^−1^, 100% NaCl rejection (membrane porosity was assumed as 10%)	Separation performance is based on MD simulation
	Nanoporous MXene	Typical range of pore size: from a few nanometers to tens of nanometers [99–101]	Simulation of a ~ 50 Å^2^ Ti-terminated pore [102]: 414 L m^−2^ h^−1^ bar^−1^, > 99% KCl rejection	Separation performance is based on MD simulation
	Nanoporous MoS_2_	Typical range of pore size: from a few angstroms to tens of nanometers [103, 104]	Simulation of a 0.74 nm pore with Mo- and S-terminated edge [105]: 178 L m^−2^ h^−1^ bar^−1^, 100% NaCl rejection (membrane porosity was assumed as 10%)	Separation performance is based on MD simulation
Assembly of materials with lamellar channels	GO nanosheets	Typical interlayer distance of GO nanosheets: 7–12 Å [106, 107] Typical interlayer distance of reduced GO nanosheets: 3.7–7 Å [108–110] Interlayer distance of graphite: 3.35 Å [111]	Simulation of GO nanosheets with 9.5 Å interlayer distance [112]: ~ 500 Lm^−2^ h^−1^ bar^−1^, 100% NaCl rejection	Separation performance is based on MD simulation The interlayer spacing could be altered by the operation parameters of filtration [113] Antibacterial property [114]
	MXene nanosheets	Typical interlayer distance of MXene nanosheets: 1.3–1.8 nm [115–117]	Simulation of Ti_3_C_2_F_2_ nanosheets with 9 Å interlayer distance [21]: ~ 3300 L m^−2^ h^−1^ bar^−1^, 100% NaCl rejection	Separation performance is based on MD simulation The interlayer spacing could be altered by the operation parameters of filtration [116] Poor stability against oxidation [118] Antibacterial property [119]
	MoS_2_ nanosheets	Typical interlayer distance of MoS_2_ nanosheets: ~ 6.2 Å [58, 120, 121] Typical interlayer distance of functionalized MoS_2_ nanosheets: 8–10 Å [58, 120, 121]	Simulation of MoS_2_-ethanol nanosheets with 5.0 Å interlayer distance [58]: 417.1 L m^−2^ h^−1^ bar^−1^, 100% NaCl rejection	Separation performance is based on MD simulation Interlayer spacing cannot be altered by operation parameters of filtration [122, 123] Poor stability against oxidation [124] Antibacterial property [125]

^a^The current table reports intrinsic material properties. The related membrane properties are presented in Online Appendix B (Table S1 for G3 membranes and Table S2 for TFN membranes)

### Porous/Non-Porous Nanofillers

A huge wave of exciting studies searching for next-generation desalination materials has surged in the new millennium [8, 29–31, 36]. These novel materials often feature intrinsic pore structures with well-defined individual channels (e.g., AQPs and CNTs) or highly porous structures (e.g., MOFs and nanoporous graphene). For example, CNTs have a hydrophobic channel that can transport water molecules in a “ballistic motion” with minimal friction [37]. Alternatively, some nanomaterials may be assembled to construct nanoscale lamellar flow channels, e.g., by the stacking of 2D nanosheets where water flows laterally between the sheets. In this section, we will provide a brief overview of the emerging generation (G3) of materials for desalination–their structures and the relevant mechanisms for desalination (Table 1). As a side note, these novel materials can also be incorporated into polyamide rejection layers as nanofillers for synthesizing so-called thin-film nanocomposite (TFN) membranes (Fig. 1c). The TFN structure, first introduced by Hoek and co-workers [38] in 2007, combines the advantages of the polyamide matrix and the nanofillers. Indeed, many novel materials–though featuring interesting pore structures–are difficult to form into a continuous separation layer, and the TFN approach provides an alternative way to utilize the intrinsic pore structures of nanofillers (e.g., for boosting membrane permeance) while maintaining membrane mechanical integrity using the polyamide matrix. Furthermore, the TFN approach also allows the use of non-porous materials, such as TiO_2_ [39, 40], Ag [41, 42], silica nanoparticles [43, 44], and graphene oxide (GO) nanosheets [45, 46]. These non-porous nanofillers could improve the separation performance by enhancing the hydrophilicity of membranes [47] or creating selective nanochannels at the filler-matrix interface [41]. In addition, some of these non-porous materials, e.g., TiO_2_ [39, 48], Ag nanoparticles [49, 50], and GO nanosheets [45], may endow membranes with additional properties, such as biofouling resistance and chlorine resistance. Nevertheless, at a fundamental level, the permeance and selectivity of TFN membranes are still constrained by the performance of the polyamide matrix. For this reason, TFN membranes can be viewed as the transitional generation (G2.5) between the TFC membranes (G2) and the next-generation RO membranes (G3) featuring the emerging desalination materials (Fig. 1).

### Individual Channels

The construction of pores or channels with tailored geometry and chemistry is the core of next-generation desalination materials. In general, these porous materials can be divided into materials with intrinsic pores and materials which form lamellar channels by assembly (Fig. 1d, Table 1). The intrinsic pores can be further classified into individual channels and multiple pores. Individual channels refer to materials that possess intrinsic channels but do not form regular continuous films by themselves. For instance, AQPs [59–61], a large family of proteins existing in biological membranes for water transport, possess a central pore of 2.8 Å in each subunit (Fig. 1d(I)). The fine pore circumscribed by hydrophobic residues only allows water molecules to go through in a single file. The combined effect of size restriction, water dipole reorientation, and electrostatic repulsion within the pore prevents the transport of species other than water. In order to mimic AQPs, AWCs constructed by simpler compounds have been investigated for desalination [62, 63]. Several kinds of AWCs have been reported, including dendritic dipeptides [64, 65], imidazole-quartet channels (I-quartets) [66, 67], pillar [5], arenes [68, 69], and polymeric foldamer-based AWCs [70, 71]. Among them, I-quartets have demonstrated promising desalination performance: when they are incorporated in a polyamide thin film, the resulting thin-film water channel (TFWC) membranes show 99.5% rejection of NaCl with a water permeance of 2.8 L m^−2^ h^−1^ bar^−1^ under seawater RO (SWRO) conditions (35,000 ppm NaCl at an applied pressure of 65 bar) [16, 17]. I-quartet channels [66], which are self-assemblies of imidazoles through noncovalent bonding, are stabilized by water-wires within the channels (Fig. 1d(II)). According to stopped-flow light scattering experiments, I-quartet channels have high water permeance and total ion rejection except for protons [67]. Analogous to AWCs, CNTs have inherent channels within the cylinders of carbon atom sheets (Fig. 1d(III)). The size [72] and functionality [73] of CNTs can influence the transport of water and ions, as shown by molecular dynamics (MD) simulations. Once water molecules enter the smooth and hydrophobic nano-channels of CNTs, the transport is frictionless [37]. CNTs with appropriate sizes could have an even higher water permeability [74] than AQPs (Table 1). Moreover, CNTs have good antimicrobial properties [75], which can benefit the biofouling resistance of membranes. Since these individual channels cannot directly form continuous mechanically stable films, they are commonly incorporated into continuous matrices that are able to resist harsh pressure conditions used for RO processes. Apart from TFN membranes with polyamide as the matrix, other continuous matrices may be adopted. For instance, the supported lipid bilayer containing AQPs or AQP-incorporated vesicles (AQP SLB membrane) [76, 77] and polymeric or inorganic matrices (e.g., polystyrene [18, 78], epoxy [28], silicon nitride [19]) filling up the spaces between nanotubes in VA-CNT have been investigated for desalination.

### Porous Crystalline Materials

Different from materials with individual channels, porous crystalline materials and porous 2D materials containing multiple channels not only can be incorporated into TFN membranes but also may form continuous films on their own, though their mechanical stability remains an important controversial issue. Porous crystalline materials, such as zeolites, MOFs, and covalent-organic frameworks (COFs), can be applied in desalination due to their high porosity and defined pore size [30, 126–130]. MD simulations show that they can achieve high water permeance and complete salt rejection (Table 1). These three porous crystalline frameworks have different structural components. Specifically, zeolites are inorganic aluminosilicates made up of SiO_4_ and AlO_4_ tetrahedra [131] (Fig. 1d(IV)). MOFs are inorganic/organic hybrid materials formed by the coordination of metal ions or clusters with organic likers [132] (Fig. 1d(V)). COFs are organic materials composed of light atoms (i.e., C, H, N, O, B) [133] connected via covalent bonds (Fig. 1d(VI)). The transport of water and ions through these porous frameworks is governed by the size of the pore and the functional groups attached to the pores [26, 126, 134–136]. The pore sizes of MOFs and zeolites are typically sub-1 nm, while COFs typically have pore sizes in the range of 1–5 nm, which are unfavorable towards water/NaCl separation. To make COFs suitable for desalination, a common strategy is to reduce their pore sizes by the addition of functional groups or a special stacking fashion (Table 1). Unfortunately, some of these materials degrade in water, such as boroxine and boronate ester-linked COFs [133] and most kinds of MOFs [137]. However, researchers have found water-stable types, for example, COFs based on imine, beta-keto-enamine, or azine linkages [138], and MOFs made by high valence metal ions [139] or imidazolate-based organic linkers [140]. These water-stable porous materials have huge potential for desalination and water treatment, subject to scalability of fabrication (see Sect. 4).

### Porous 2D Materials

Several 2D materials with multiple nanoscale pores also show good potential for desalination. The most famous example of 2D materials is graphene, a single layer of sp^2^-bonded carbon atoms [141, 142]. Since graphene is impermeable to water, nanoscale pores could be created onto graphene by oxygen plasma or ion bombardment to prepare nanoporous graphene [96, 97, 143, 144] (Fig. 1d(VII)). Nanoporous graphene is ideal for desalination because of its one-atomic ultra-thin thickness, which can facilitate high water permeance. Meanwhile, graphene has outstanding antifouling properties and high chlorine tolerance [114, 145], both of which are advantages for next-generation RO membranes. Apart from nanoporous graphene, nanoporous MXene and nanoporous MoS_2_ have also been investigated for desalination. MXenes are early transition metal carbides and/or carbonitrides [57, 146] (Fig. 1d(IX)). They have graphene-like morphology, hence the name MXenes [57]. MoS_2_ is a layered metal chalcogenide composed of one sheet of Mo atoms sandwiched between two sheets of S atoms [58] (Fig. 1d(X)). Both single-layer MXene and single-layer MoS_2_ can be made by exfoliation [57, 121, 147, 148]. Nevertheless, the investigation of nanoporous MXene and nanoporous MoS_2_ for pressure-driven desalination is still in the stage of simulation (Table 1). In principle, ultra-thin 2D MOFs and 2D COFs can also be classified as porous 2D materials.

### Assembly of Materials with Lamellar Channels

In contrast to materials with intrinsic pores, nanosheets of 2D materials, such as GO [149, 150] (Fig. 1d(VIII)), MXene [117, 151] (Fig. 1d(IX)), MoS_2_ nanosheets [58, 121] (Fig. 1d(X)), can form lamellar channels by assembly/stacking. The lamellar channels in these stacked 2D materials allow water to flow through and retard hydrated ions and other solutes. Therefore, the interlayer distance and the surface functional groups of these 2D nanosheets regulate the separation performance of these membranes [21, 112, 152, 153]. Table 1 provides a comparison of these 2D materials on the basis of MD simulations. MoS_2_ nanosheets have stable interlayer spacing because of the absence of hydrophilic groups and strong van der Waals forces between layers [122, 123]. In contrast, the interlayer distance of GO nanosheets and MXene nanosheets are often altered by operation parameters of filtration, such as pH [154, 155], pressure [156], and solute concentration of feed solutions [113, 115]. To mitigate this issue, intercalation with high-valent metal ions (e.g., Al^3+^ [116]) and crosslinking via covalent bonds [22] have demonstrated some degree of success. It is also worthwhile to note that MoS_2_ [124] and MXenes [118] can easily get oxidized in ambient conditions, which could limit their practical applications.

## Separation Performance of RO Membranes

**Fig. 2:**
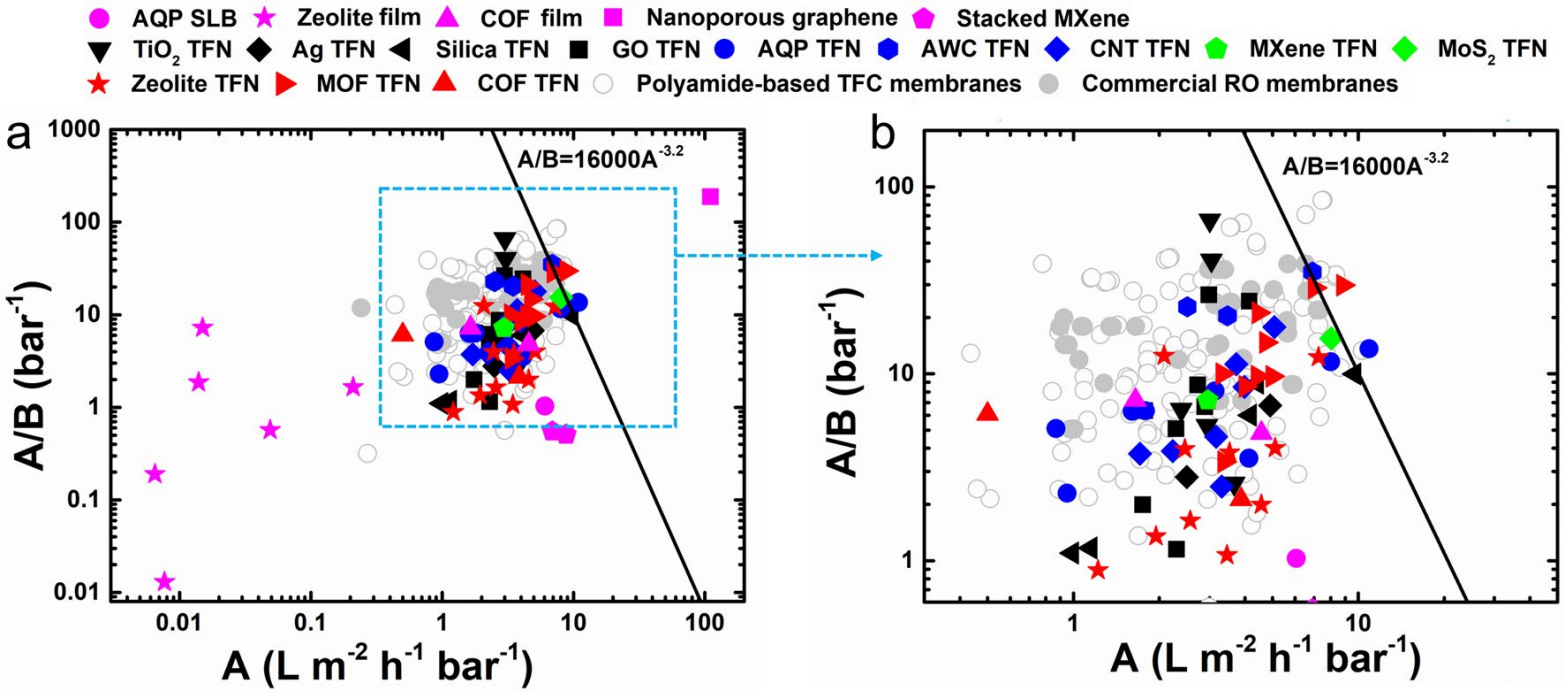
Permeance and water/NaCl selectivity of **a** RO membranes and **b** TFN membranes. More details of the calculation of *A/B* and *A* are provided in Online Appendix A. Data of *A* and *A/B* for novel RO membranes were collected from literature (Online Appendix B). For comparison, the data for lab-made polyamide-based TFC membranes obtained from the Open Membrane Database [158] accessed on October 26th, 2024, with “Polyamide”, “Polymeric TFC”, and “No modification” as filters (empty light grey symbols), the data for commercially available RO membranes [6] (solid light grey symbols), and the “2019 upper bound” of TFC membranes for desalination [6] (black line) have been included in the figure. Membranes without well-established data of *A/B* and *A* (e.g., VA-CNT, MOF thin film, stacked GO, and stacked MoS_2_) were not included

Water permeance and selectivity are two key indicators for RO membrane performance. To evaluate membrane performance, a plot of water/NaCl permselectivity (*A/B*) versus water permeance (*A*) is adopted following the approach of Yang et al. [6, 7]. *A/B* and *A* are preferred over intrinsic water/NaCl permeability selectivity (*P*_*w*_*/P*_*s*_) and intrinsic water permeability (*P*_*w*_) because *P*_*w*_ and *P*_*s*_ are dependent on membrane thickness that is often unavailable or inaccurately measured in many published papers. In addition, the water permeance *A* value better reflects the available water flux under a given pressure driving force. Figure 2 summarizes the separation performance of RO membranes made of various novel materials. For benchmarking purposes, data points for conventional lab-made polyamide-based TFC membranes (empty light grey symbols) and commercially available RO membranes (solid light grey symbols) are included. Furthermore, the “2019 upper bound” (black line in Fig. 2) [6], representing the highest performance of TFC membranes, is also superimposed in Fig. 2. In general, TFN membranes with various nanofillers show similar or sometimes slightly better separation performance compared with existing TFC polyamide membranes and the “2019 upper bound” (Fig. 2b). As discussed in Sect. 2, these G2.5 membranes generally rely on the polyamide matrix to maintain membrane integrity (and thus to minimize membrane defects), such that their separation performances are strongly influenced by the polyamide backbone. At the same time, TFN membranes offer opportunities for permeance/selectivity enhancement by taking advantage of the raised permeability of the intrinsic pore structures of nanofillers or interfacial selective channels induced by the nanomaterials [41]. Enhancing the incorporation density of the nanomaterials within the polyamide backbone and the alignment of nanochannels (e.g., CNTs [157]) to facilitate the transport of water molecules could lead to an additional improvement in the performance of TFN membranes.

Surprisingly, many G3 membranes, made of novel materials without a polyamide matrix, do not appear to offer competitive separation performances in pressure-driven desalination experiments (Fig. 2). Indeed, the experimental results reported in the literature are often far below the theoretical predictions (Table 1). This mismatch can be ascribed to the thickness and/or defects in these novel membranes. For example, zeolite membranes, hampered by their micrometer-level thickness [159–163] (in contrast to 10–20 nm for RO polyamide layers [164]), generally have low water permeance (< 0.5 L m^−2^ h^−1^ bar^−1^) (Fig. 2a). Their available selectivity is also moderate (< 10 bar^−1^), which is not in consonance with the theoretical NaCl rejection derived from simulation [84] (Table 1). Similarly, AQP SLB membranes and stacked MXene membranes commonly present low selectivity (Fig. 2a) due to their unavoidable defects [165–169]. COF membranes have low selectivity (Fig. 2a) not only because their typical pore size is in the range of nanofiltration/ultrafiltration [138] but some defects and amorphous regions can harm selectivity [129, 169]. Even though the selectivity of COF membranes can be theoretically boosted by some special stacking fashions [90] or uniform functionalization [89, 91], it often cannot be easily achieved in practice. Nevertheless, it is also worthwhile noting some exceptional cases of G3 membranes, e.g., nanoporous graphene with optimal pore size supported by a single-walled CNT network [20]. This membrane offers a thin and defect-free structure, demonstrating extremely attractive separation performance (Fig. 2a) that is in accordance with the simulation results [98, 170] (Table 1). The salient example of nanoporous graphene demonstrates the huge potential of the next-generation G3 membranes for simultaneously boosting permeance and selectivity, provided that membrane thickness and defect formation can be well controlled.

## Multi-Dimension Evaluation of Novel RO Membranes

**Fig. 3:**
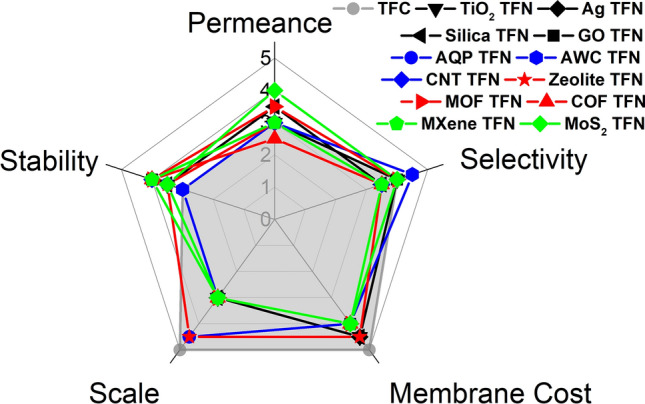
Radar chart for TFN membranes. As a benchmark, conventional TFC membranes are represented by the grey polygon (based on typical BWRO properties). Details for the evaluation and rubrics are provided in Table S2 and Online Appendix C, respectively. Separate radar charts for each TFN membrane are provided in Fig. S3 (Online Appendix D)

**Fig. 4:**
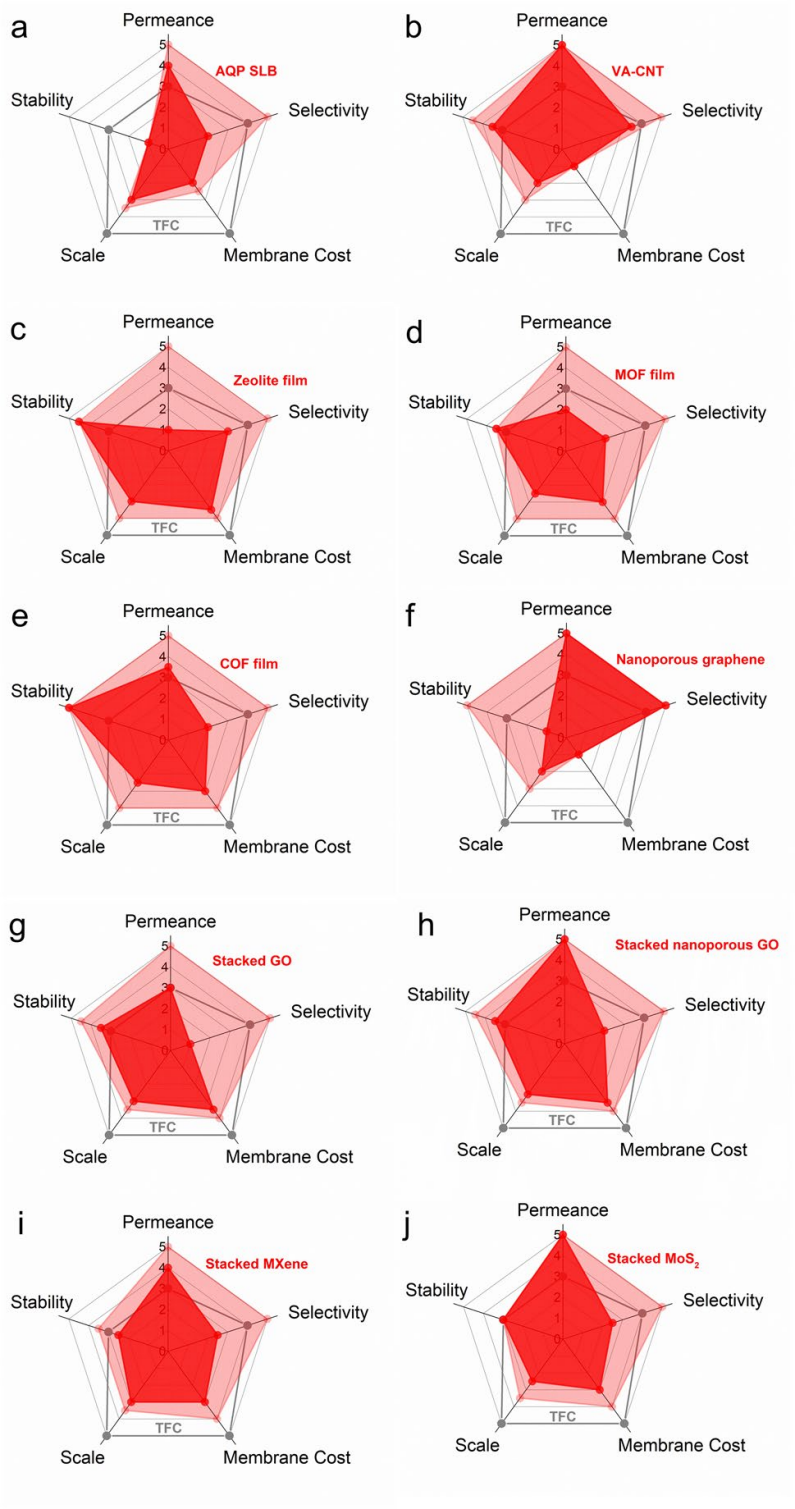
Radar charts for the multi-dimension evaluation of **a** AQP SLB membrane, **b** VA-CNT membrane, **c** zeolite film, **d** MOF film, **e** COF film, **f** nanoporous graphene membrane, **g** stacked GO membrane, **h** stacked nanoporous GO membrane, **i** stacked MXene membrane, and **j** stacked MoS_2_ membrane. The status and potential of novel RO membranes are represented by the dark-red and light-red regions, respectively. The status of conventional TFC membranes is represented by the empty grey polygon as a benchmark. Details for the evaluation and rubrics are provided in Table S1 and Online Appendix C, respectively

To facilitate the application of novel RO membranes, different dimensions of membranes should be considered. Apart from the basic separation performance, additional aspects, including membrane cost, scale, and stability, could significantly affect their commercialization. For example, although nanoporous graphene, described above, has outstanding separation performance [20], its high fabrication cost, difficulty in scaling up, and poor mechanical properties could be decisive factors limiting its commercial success [171, 172]. Therefore, a systematic re-evaluation of literature demonstrating the strengths, weaknesses, and potential of novel RO membranes is necessary to figure out their development directions. In this section, five dimensions (i.e., permeance, selectivity, membrane cost, scale, and stability) are scored in radar charts to provide a holistic evaluation of novel RO membranes (TFN membranes in Fig. 3 and various G3 membranes in Fig. 4), with a higher score (on a scale of 5) indicating a better membrane attribute according to the detailed rubrics in Online Appendix C.

### TFN Membranes

Figure 3 evaluates TFN (G2.5) membranes fabricated using different novel materials. The currently available permeance and selectivity are scored based on the value of *A* and *A/B* (or NaCl rejection if *A/B* is not available) of TFN membranes tabulated in Table S2 together with the rubrics in Online Appendix C. Conventional TFC (G2) membranes are represented by the grey-shaded polygon in the same figure for benchmarking purpose. Based on their typical water permeance (1–5 L m^−2^ h^−1^ bar^−1^) and water/NaCl selectivity (5–30 bar^−1^), the state-of-the-art TFC membranes are scored 3 for permeance and 4 for selectivity. TFN membranes can achieve slightly improved separation performance compared to TFC membranes due to the intrinsic pore structures of nanofillers or interfacial selective channels induced by nanomaterials in the polyamide matrix [41]. However, the dispersion of nanofillers in polyamide matrices can be a concern since the aggregation of nanofillers may result in defects that hamper membrane selectivity [173]. For example, in I-quartet-based TFWC membranes, the assembly of I-quartet on the substrate before the IP process [16] is a challenging step, which may significantly affect the separation performance. To better disperse nanofillers, it is crucial to carefully choose the dispersion solution (aqueous vs. organic solution), nanofiller concentration, and surface modification of nanofillers (e.g., polydopamine (PDA) coating to increase the hydrophilicity of CNTs [157]). In addition, in-situ fabrication of TFN membranes could be a promising strategy to uniformly distribute nanofillers without requiring additional processes for nanomaterial synthesis (e.g., Ag nanoparticles reduced from AgNO_3_ by *m*-phenylenediamine (MPD) [42] and silica nanoparticles polymerized from tetramethoxysilane [43]).

Figure 3 also systematically evaluates additional dimensions of TFN membranes in comparison with TFC membranes (see detailed rubrics in Online Appendix C). Membrane cost is scored based on the material fabrication and membrane synthesis (Fig. S2), and the scale of the current development is scored based on the reported membrane area (Table S2). Meanwhile, the stability score reflects the overall considerations of mechanical stability, thermal stability, chemical stability, and fouling resistance of membranes (Tables S3 and S4). Conventional TFC membranes, serving as the benchmark, receive a full score of 5 with respect to both cost and scale of development due to their mature commercialization and large-scale applications worldwide. On the other hand, their poor chlorine resistance [10, 11] and high fouling propensity [13, 14] are responsible for the relatively low score of 3 with respect to stability. Since TFN membranes typically adopt a polyamide matrix, their chemical and thermal stability would be largely constrained by those of the polyamide material. Nevertheless, some nanofillers could potentially enhance antifouling performance [17, 41] or chlorine resistance [174, 175], thereby leading to slightly improved scores for stability in the corresponding TFN membranes (Table S2). In terms of cost, TFN membranes are often slightly more expensive due to the additional costs associated with nanofillers. Nevertheless, the typical low dosage of nanofillers does not appear to be a major obstacle to their commercialization. To date, several types of TFN membranes have already been commercialized, such as LG Chem’s NanoH_2_O™ [176] and Aquaporin Inside® membranes [177], incorporating zeolites and AQPs as nanofillers, respectively. Therefore, these TFN membranes are scored favorably with respect to the scale of development. Many other types of TFN membranes, such as MXene TFN and MoS_2_ TFN membranes, are still at bench scale (Table S2), and their full-scale production has yet to be demonstrated.

For large-scale applications, some commercial TFN membranes (e.g., AQP TFN [177] and zeolite TFN [176]) have already been used in water treatment processes such as desalination, wastewater treatment, and water purification to efficiently remove salts and other impurities from water [176, 178]. However, for some emerging TFN membranes that involve expensive or poorly-dispersed nanofillers, fabricating a standard spiral wound module with a relatively large membrane area (~ 40 m^2^ [179]) is still a daunting challenge. Nevertheless, such TFN membranes might still find niche applications that demand a relatively small membrane area (e.g., biomedical applications such as drug delivery [180]). Another challenge for TFN membranes is the potential leaching of nanomaterials, which can negatively impact the life span of membranes [47]. The leached nanomaterials may also cause toxicity to aquatic organisms [181, 182], raising potential threats to ecology and human health [183].

Another interesting example of TFN is TFWC membranes containing I-quartet water channels (Online Appendix D, Fig. S4). With optimally dispersed densely packed AWCs within a polyamide matrix, the resulting TFWC-RO biomimetic membrane provides an apparent NaCl rejection of 99.5% and with a water flux of 75 L m^−2^ h^−1^ at SWRO testing conditions, i.e., 65 bar applied pressure with 35,000 ppm NaCl [16]. This corresponds to a water permeance of 2.5 L m^−2^ h^−1^ bar^−1^, an intrinsic NaCl rejection of 99.8%, and a water/NaCl selectivity of 22.8%, which is far better compared to the control TFC polyamide membrane without the inclusion of I-quartets. Indeed, this water permeance is comparable to some brackish water RO (BWRO) membranes and far superior to commercial SWRO membranes (~ 1 L m^−2^ h^−1^ bar^−1^). At the same time, its NaCl rejection is as good as that of typical SWRO membranes. The combination of these separation properties makes the TFWC membrane a favorable candidate compared to both SWRO and BWRO (Online Appendix D, Fig. S4). This TFWC membrane shows remarkable mechanical stability, making it a good candidate for both SWRO desalination and water reuse applications. On the other hand, its chemical stability (e.g., chlorine resistance and pH stability) would be largely constrained by its polyamide matrix. To date, the production of TFWC membranes is still at the bench scale, resulting in a relatively low score of 3 with respect to the scale of development. Nevertheless, since most of the fabrication procedures are compatible with commercial TFC production lines, large-scale production of TFWC membranes should be feasible at a cost slightly higher than their TFC benchmarks. In addition, the separation performance and stability of TFWC might be further enhanced to better unleash the intrinsic material properties of AWCs, provided that a more suitable matrix can be developed to overcome the current limitations of polyamide. Similar future potential developments are also applicable to other TFN membranes shown in Fig. 3.

### Novel G3 Membranes

Figure 4a-j systematically benchmarks different novel G3 RO membranes against conventional TFC membranes (shown as the empty grey polygon). To differentiate their current development status and the ultimate potential, we adopt the dark-red region to represent the current state (based on available experimental data) and the light-red region to show the fundamental limits (based on theory and simulation) for each G3 membrane type. For example, in contrast to the currently available permeance and selectivity that are scored based on experimental membrane performance (Table S1), the corresponding ultimate potentials are scored based on the theoretical performance of materials (Table 1). Since all the novel materials listed in Table 1 show highly attractive intrinsic separation properties, they receive scores of 5 for both theoretical permeance and theoretical selectivity. Therefore, the differences between the currently reported membrane separation performance and the ultimate material potential reveal the critical development gaps. Similarly, while the current scale of development is evaluated based on the experimentally fabricated membrane area (Table S1), the ultimate potential in scaling up is scored according to the difficulty level of fabrication techniques.

**Fig. 5:**
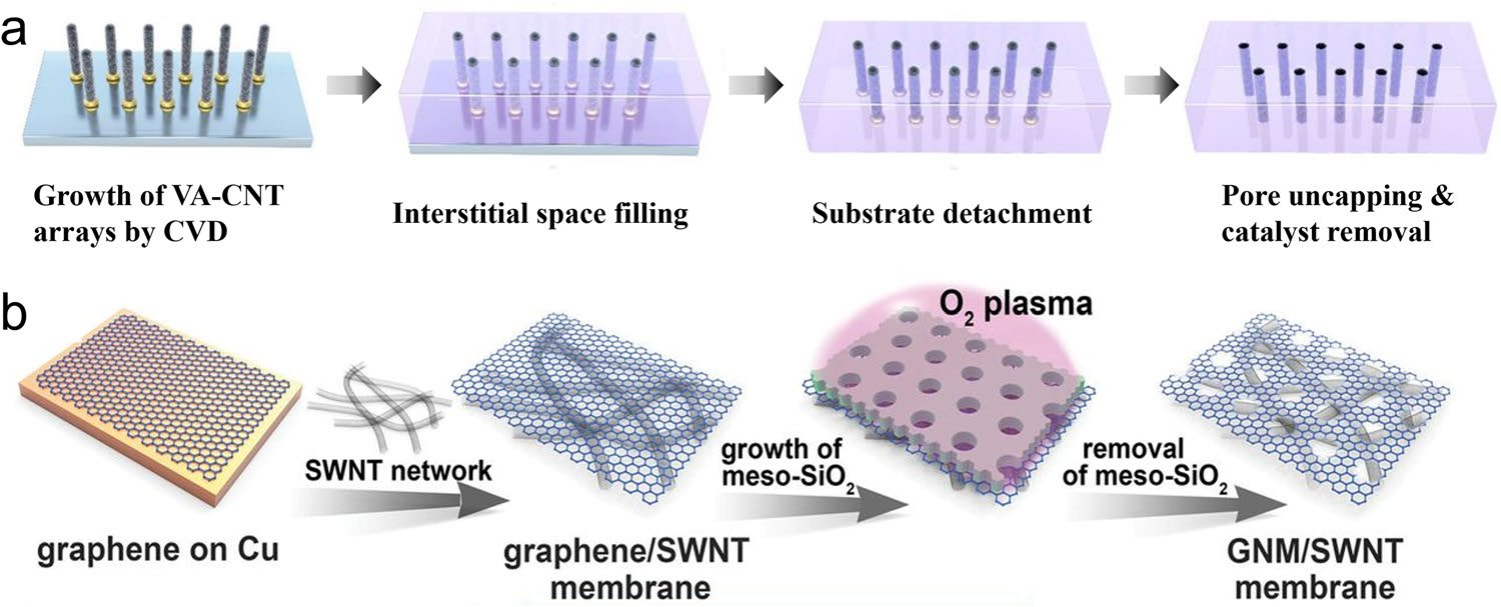
Schematic illustration of the fabrication process of **a** VA-CNT membrane adapted from Ref. [187]; **b** nanoporous graphene, reproduced from Ref. [20], copyright © 2019, The American Association for the Advancement of Science

The radar charts reveal certain Achilles’ heels that can restrict the practical applications of many G3 membranes. For instance, despite the high water permeance and potentially high selectivity of AQP SLB membranes, they have problematic stability issues (Fig. 4a) due to the mobility and potential degradation of the lipid layer and the denaturation of proteins [76, 77]. The demanding fabrication process for these membranes, involving expression and purification of AQPs, preparation of proteoliposomes, and vesicle rupture [80, 184, 185], further causes a low score for membrane cost and scale of development. Similarly, the high fabrication cost is the main constraint for many other G3 membranes, such as nanoporous graphene (Fig. 4f) and VA-CNT membranes (Fig. 4b). These membranes typically involve CVD or other complex procedures in their fabrication processes (Fig. 5), leading to high membrane cost and limited scale of development. Therefore, developing more scalable and cost-effective fabrication strategies is critically needed for such membrane development. Potential revolutions in fabrication methods, e.g., replacing CVD deposition of CNTs by filtration-based loading for VA-CNT membranes or by emerging 3D printing strategies [186], might dramatically reduce the membrane cost and promote their future scale-up. In addition to cost and scale-up, practical applications of ultra-thin nanoporous graphene could be further restricted by its poor mechanical strength [171, 172]. Addressing this mechanical weakness issue, e.g., by designing advanced supporting structures, might greatly improve the stability score of nanoporous graphene, particularly in view of its tolerance for high-temperature feed water, chlorine attack, and acidic or basic solutions. Therefore, the critical constraints revealed by these radar charts could be used to prioritize future research efforts to make the respective membranes more competitive.

**Fig. 6:**
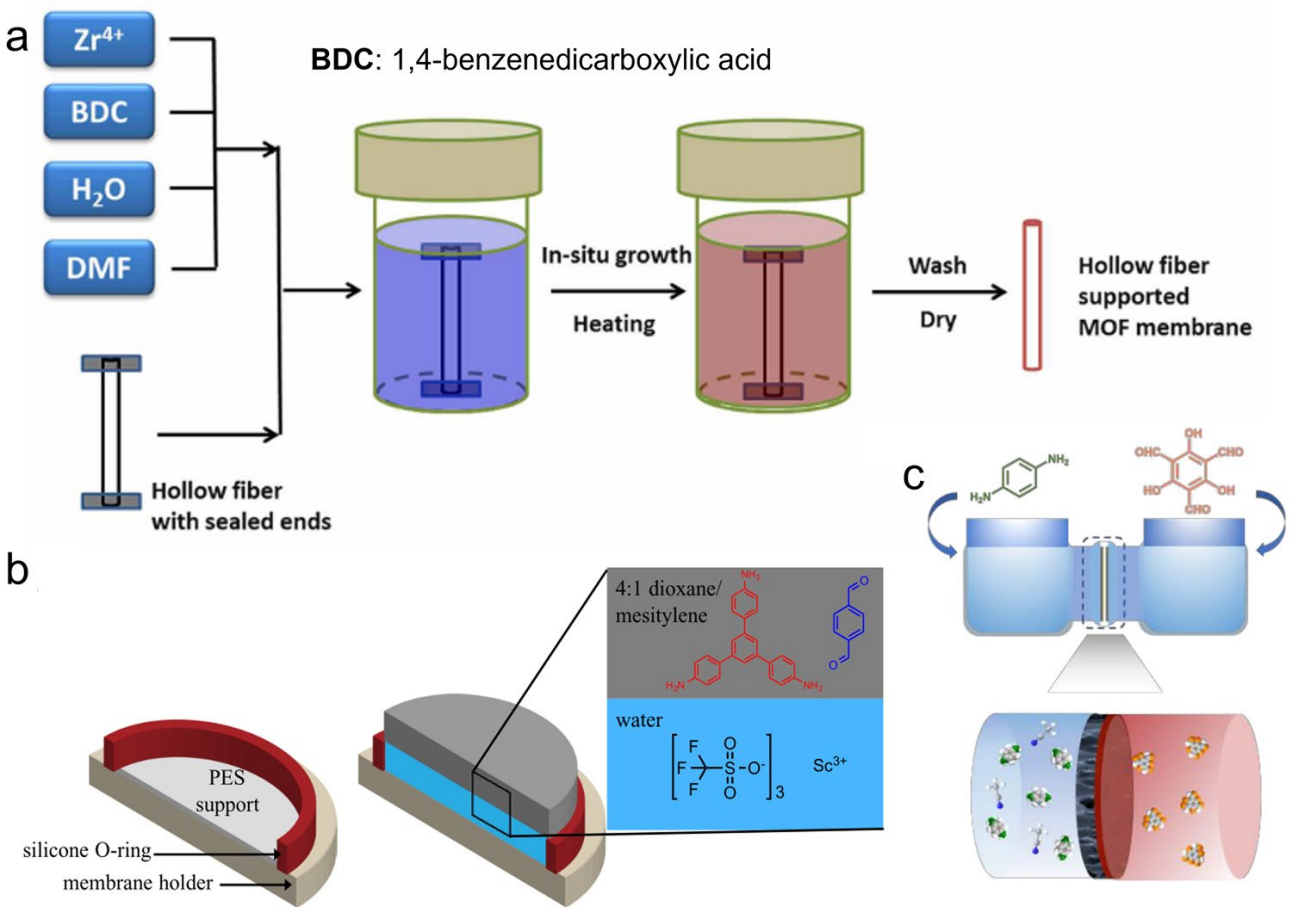
Schematic illustration of the fabrication of **a** MOF film by an in-situ solvothermal method, reproduced with permission from Ref. [139], copyright © 2015, American Chemical Society; **b** COF film by an IP reaction, reproduced with permission from Ref. [193], copyright © 2017, American Chemical Society; **c** COF film by counter-diffusion, reproduced with permission from Ref. [169], copyright © 2022 Elsevier B.V

In the radar charts, G3 membranes made of porous crystalline materials (i.e., zeolite film, MOF film, COF film, Fig. 4c-e) represent an interesting group. These membranes can be typically fabricated by a range of relatively simple methods, paving the way for future scale-up. Specifically, zeolite films can be synthesized by seeded assembly and secondary growth [188]; MOF films can be fabricated by in-situ solvothermal synthesis [139, 189, 190] or secondary seeded-growth [127, 191, 192]; COF films can be made by polymerization at free-interface [193, 194], counter-diffusion [169, 195], or secondary growth [196] (Fig. 6). In addition, they can be incorporated onto porous inorganic hollow fibers or tubes [139, 161–163, 187, 191, 192, 197], which are also beneficial for their scaling up. Moreover, their mechanical problems can be improved by compositing with suitable substrates. For example, ultra-thin COF films [198, 199] can be supported by polyacrylonitrile (PAN) [194, 200], anodic aluminium oxide (AAO) [201], polysulfone (PSF) [202] substrates, etc. With a proper choice of material (Tables 1 and S1, S4), these porous crystalline membranes could also offer good thermal and chemical stability and fouling resistance. For example, zeolite films can withstand high temperatures as high as 80 °C [203]. COF films, benefiting from their covalent bonds, can also be thermal-stable and pH-stable [196, 204–207] under harsh operational conditions. Additionally, in contrast to the poor chlorine resistance of typical polyamide-based membranes, many of these porous crystalline membranes are stable in NaClO solution [175, 188, 208], which is advantageous for membrane (bio)fouling control and cleaning. It is interesting to note that, despite their excellent intrinsic separation properties shown in Table 1, the state-of-the-art membranes made of zeolite, MOF, and COF films generally show limited water permeance and water/salt selectivity under typical pressure-driven membrane tests. For example, UiO-66 membrane has a permeance of 0.14 L m^−2^ h^−1^ bar^−1^ and a NaCl rejection < 50% [139], far below its theoretical performance (a permeance of 51 L m^−2^ h^−1^ bar^−1^ and a NaCl rejection of 100% [86]). This huge gap in separation performance is often caused by the relatively high thickness of rejection layers and defects in the membranes. If these issues could be tackled, zeolite/MOF/COF-based porous crystalline G3 membranes could potentially perform well in all five dimensions—serving as pentagon warriors for next-generation desalination membranes. Therefore, to fully realize their potential, more research efforts should be put into the manipulation of the thickness, pore size, defects, and framework flexibility of these membranes [25, 209–212].

**Fig. 7:**
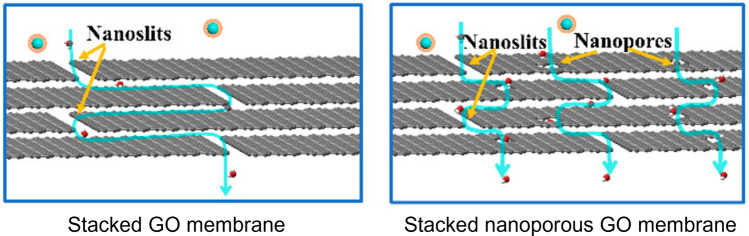
Schematic illustrations of the water transport pathways in the stacked GO and stacked nanoporous GO membranes, adapted with permission from Ref. [108], copyright © 2019, American Chemical Society

2D materials such as GO, MXene, and MoS_2_ represent another category of competitive candidates for synthesizing next-generation desalination membranes. These materials can be easily vacuum-filtrated onto a porous substrate to prepare membranes featuring stacked 2D nanosheets. Many stacked 2D membranes have high chlorine tolerance [116, 120]. Nevertheless, for MXenes and MoS_2_, their oxidation, even under ambient conditions, could be a critical barrier to their commercialization. In addition, stacked 2D membranes show limited interlayer stability: their interlayer spacing can be altered by pressure [156] and solution chemistry during the filtration process [113, 115, 116, 154, 155]. The adhesion between 2D nanosheets and substrates is another concern. To address these issues, different crosslinking strategies have been applied to stabilize the interlayer distance [116, 117, 120, 154, 156] and/or to improve the adhesion between the 2D materials and the substrates [213]. For separation properties, existing stacked GO membranes (Fig. 4g) could achieve comparable (or even potentially better) water permeance compared to conventional TFC membranes (Table S1). Stacked MXene (Fig. 4i) and stacked MoS_2_ (Fig. 4j) membranes also exhibit high water permeance. Nevertheless, stacked 2D membranes commonly suffer low water/salt selectivity due to defects and large interlayer distances [113, 121, 151, 153, 154, 156, 168]. In order to further improve the separation properties of stacked 2D membranes, one potential strategy is to adopt 2D nanosheets containing selective pores, which improve the transport of water molecules while retaining solutes. For instance, stacked nanoporous GO membranes (Fig. 4h) provide more water transport pathways and shorten their transport distance (Fig. 7), leading to simultaneously increased selectivity and water permeance [108, 214] in comparison with the stacked GO without nanopores (Fig. 4g). Similarly, 2D MOF and COF nanosheets with high porosity [215, 216] are also competitive candidates for fabricating high performance stacked 2D membranes.

## Conclusions and Outlook for Future Development of RO Membranes

This review provides a comprehensive summary and systematic evaluation of RO membranes made by novel materials, including both (1) transitional generation (G2.5) TFN membranes that incorporate novel materials into a polyamide matrix and (2) next-generation (G3) membranes featuring emerging materials without a polyamide matrix. Despite the superior separation properties for many materials, the reported separation performance of corresponding membranes (in the form of an *A/B* versus *A* upper-bound plot) is often not ideal when benchmarked against existing conventional TFC membranes, highlighting critical development gaps, particularly with respect to defect management. The defects of TFN membranes commonly arise from the aggregation of nanofillers, which can be partially mitigated by proper surface modification of nanofillers [157]. In addition, some recent studies [42, 43] also report the in-situ formation of nanofillers as an effective strategy to overcome this issue. For G3 membranes, potential strategies to mitigate defects and increase stability include the use of crosslinkers [116, 213], enhanced crystallization via thermal treatment [159, 161], and surface coating for sealing defects [168]. We further established a comprehensive framework, adopting five dimensions, including stability, cost, and scalability, in addition to water permeance and selectivity, for evaluating the present development and future potential of these novel membranes. The state-of-the-art TFN membranes are competitive in all dimensions, yet their ultimate performance is generally limited by their polyamide matrix. On the other hand, many G3 membranes could be greatly constrained by their problematic stability, high cost, and/or poor scalability (e.g., AQP SLB membrane, VA-CNT membrane, nanoporous graphene). These critical deficiencies revealed by 5-dimensional radar charts require revolutionary technique changes (e.g., filtration-based loading for VA-CNT membranes in the replacement of CVD deposition and design of suitable supporting structures for nanoporous graphene [144]) for further development of these membranes. Among G3 membranes, porous crystalline membranes (i.e., zeolite film, MOF film, and COF film) are advantageous in their scale-up and stability but require research efforts (e.g., optimization of reaction conditions to reduce membrane thickness) to improve their practical water permeance and water/salt selectivity. Stacked 2D membranes are deficient in their stability and water/salt selectivity. Their stability may be enhanced by different crosslinking strategies, while their selectivity can be potentially improved by the introduction of selective pores.

The current work largely focuses on RO membranes for desalination, with a key emphasis on separation performance with respect to water permeance and salt removal. It is important to note that the competitiveness of novel membranes will depend on the application scenarios. For example, membranes with high water permeance offer few benefits for highly saline feedwater whose energy consumption is dictated by the transmembrane osmotic pressure [217–220], yet they can greatly reduce energy consumption when treating low-salinity feedwater [7, 217]. Ultra-permeable membranes (with a permeance of 50–100 L m^−2^ h^−1^ bar^−1^) may even enable new process development, such as vacuum-driven submerged RO/nanofiltration [217, 221], and their hollow fiber module configurations could be potentially adopted to enhance membrane packing and improve mass transfer over traditional spiral wound modules. Therefore, it is important to consider membranes, processes, and applications in a holistic manner to fully realize the benefits of next-generation membranes, and application-specific weighting factors may be applied to relevant dimensions for the selection of the most desirable membranes/materials. Indeed, membranes have been widely used far beyond desalination, which may require tailored properties (e.g., high Li^+^/Mg^2+^ selectivity for lithium extraction from salt lakes [222, 223], high solvent resistance and solvent permeance for organic solvent filtration [224, 225]). Many G3 membranes, even though they may not be competitive for desalination, may offer great advantages in other applications. For example, membrane cost may be less concern in some niche and high-value-added applications such as hemodialysis [180], batteries and fuel cells [226, 227], or even recycling water in space stations [228]. The comprehensive framework presented in the current work could offer holistic evaluation and benchmarking for future membrane development and may be further extended/adapted to cover more materials and a wide range of applications.

## Supplementary Information

Below is the link to the electronic supplementary material.

Supplementary file1 (DOCX 1762 KB)

## Abbreviations

2D: Two-dimensional
AAO: Anodic aluminium oxide
AQPs: Aquaporins
AQPZ: Aquaporin-Z
AWCs: Artificial water channels
BSA: Bovine serum albumin
BWRO: Brackish water reverse osmosis
CNTs: Carbon nanotubes
COFs: Covalent-organic frameworks
CVD: Chemical vapor deposition
G1: First-generation
G2: Second-generation
G2.5: Transitional generation
G3: Emerging generation
GO: Graphene oxide
I-quartets: Imidazole-quartet channels
IP: Interfacial polymerization
MD: Molecular dynamics
MOFs: Metal–organic frameworks
MPD: *m*-Phenylenediamine
PAN: Polyacrylonitrile
PAPs: Peptide-appended pillar[5]arenes
PDA: Polydopamine
PEI: Polyetherimide
PES: Polyethersulfone
PDMS: Polydimethylsiloxane
PVA: Polyvinyl alcohol
PSF: Polysulfone
RO: Reverse osmosis
SLB: Supported lipid bilayer
SWRO: Seawater reverse osmosis
TFC: Thin-film composite
TFWC: Thin-film water channel
TFN: Thin-film nanocomposite
TMC: Trimesoyl chloride
VA-CNT: Vertically aligned CNT
