# Supplementary material for: Next-Generation Desalination Membranes Empowered by Novel Materials: Where Are We Now?
Source: Nanomicro Lett. 2024 Dec 20;17:91. doi: 10.1007/s40820-024-01606-y (PMC11659558; doi:10.1007/s40820-024-01606-y)
Supplement: Supplementary file 1 — Supplementary file1 (DOCX 1762 KB) [file 40820_2024_1606_MOESM1_ESM.docx]

Supporting Information for

**Next-Generation Desalination Membranes Empowered by Novel Materials: Where Are We Now?**

Siqi Wu^1^, Lu Elfa Peng^1^, Zhe Yang^1^, Pulak Sarkar^1^, Mihail Barboiu^2^, Chuyang Y. Tang^1,^*, Anthony G. Fane^3^

^1^ Department of Civil Engineering, The University of Hong Kong, Pokfulam, Hong Kong SAR, P. R. China

^2^ Institut Européen des Membrane, University of Montpellier, ENSCM, CNRS UMR5635, Place Eugène Bataillon, CC 047, 34095 Montpellier, France

^3^ UNESCO Centre for Membrane Science and Technology, School of Chemical Engineering, The University of New South Wales (UNSW), Sydney, NSW, 2052, Australia

*Corresponding author. E-mail: [tangc@hku.hk](mailto:tangc@hku.hk) (Chuyang Y. Tang)

**Appendix A: Calculation of A/B and A**

Water permeance *A* (also called water permeability coefficient) and solute permeability coefficient *B* were calculated based on the solution-diffusion model [S1, S2] by the following equations:

$A=J_{w}/(\Delta P-{\Delta\pi}_{b}f_{cp})$ (*S1*)

$B=J_{w}*C_{p}/({\Delta C}_{b}*f_{cp})$ (*S2*)

where *J_w_* is the water flux, *ΔP* is the difference of hydraulic pressure across the membrane, *Δπ_b_* and *ΔC_b_* are the differences in osmotic pressure and concentration between the bulk feed water and the permeate water, respectively, and *C_p_* is the concentration of solute in the permeate. The modulus of concentration polarization, *f_cp_*, can be calculated by the following equation:

$f_{cp}=exp(J_{w}/K)$ (*S3*)where *K* is the mass transfer coefficient of solute (assumed to be 100 L m^-2^ h^-1^ if the exact value is not available [S3]).

It is worthwhile to note that *A/B* and *A* were calculated based on pressure-driven filtration experiments. Data from forward osmosis, distillation, pervaporation, and electrodialysis were not used due to different driving forces for separation.

**Appendix B: Research studies of novel membrane materials**

**Table S1** Research studies of novel membrane materials

|  | **Fabrication method** | ***A* (L m^-2^ h^-1^ bar^-1^)** | ***A/B* (bar^-1^) or selectivity related information** | **Test conditions** | **Fabricated (F) and/or tested (T) membrane area** | **Stability** | **Refs.** |
| --- | --- | --- | --- | --- | --- | --- | --- |
| **AQP SLB** | AQPZ-incorporated lipid vesicles with positive charges were deposited on the polyelectrolytes membrane (prepared by layer-by-layer assembly) surface. | 6.06 | 1.03 | Cross-flow (20 cm s^-1^), 0.5 g L^-1^ NaCl, 4 bar | 19.56 cm^2^ (T) | The membrane operated stably for at least 36 hours. Triton X-100 treatment slightly influenced the flux and salt rejection of the membrane. The membrane had decreased flux and rejection when stored in phosphate-buffered saline for seven days, probably due to the degradation of the SLB. | [S4] |
|  | AQPZ-incorporated triblock polymer vesicles with disulfide functional groups were spread onto gold-coated solid substrates by covalent bonding. | 16.1 ± 3.3 | 45.1 ± 4.2% NaCl rejection with a permeance of 8.2 ± 5.1 L m^-2^ h^-1^ bar^-1^ | Stirred dead-end (600 rpm), 5 bar, 0.2 g L^-1^ NaCl, 22 ± 1 ℃ | 0.2 cm^2^ (T) |  | [S5] |
|  | AQPZ-incorporated triblock polymer vesicles were ruptured and UV polymerized to form a selective layer on a methacrylate-functionalized cellulose acetate substrate. | 34.19 ± 6.90 | 32.86 ± 9.12% NaCl rejection | Stirred dead-end, 5 bar, 0.2 g L^-1^ NaCl, 22 ± 1 ℃ | 7.1 mm^2^ (T) |  | [S6] |
|  | AQPZ-incorporated proteoliposomes were coated by PDA and deposited onto a poly(amide-imide) substrate. The proteoliposomes were crosslinked with the substrate through PEI. |  | 95.0 ± 0.70% MgCl_2_ rejection with a flux of 36.6 ± 2.2 L m^-2^ h^-1^ | Stirred dead-end, 1 bar, 0.1 g L^-1^ MgCl­_2_, 21.9 ℃ | 28.26 cm^2^ (F) | AQPZ maintained its activity at 343 K for 2 hours. | [S7] |
|  | The lipid bilayer containing AQPZ was covalently bonded to the PDA-coated PSF substrate by an amidation reaction. |  | 90% MgCl_2_ rejection with a flux of 25.3 L m^-2^ h^-1^ | Dead-end, 4 bar, 2 g L^-1^ MgCl_2_ | 19.6 cm^2^ (T); 36 cm^2^ (F) | Covalent bonding enhanced membrane stability. The membrane had slightly decreased flux and rejection when stored in phosphate-buffered saline for nine days. | [S8] |
| **VA-CNT** | CNTs were vertically aligned by CVD and incorporated into polystyrene. The substrate was etched by HF acid. The excess polymer on the surface was removed by plasma oxidation to open the CNT tips. The membrane was functionalized with biotin and coordinated by streptavidin. |  | The ionic flow was nearly blocked. | Diffusion, 1.5 g L^-1^ Ru(NH_3_)_6_Cl_3_ | 4 cm^2^ (F); 0.028 cm^2^ (T) |  | [S9] |
|  | Aligned multi-walled CNTs were incorporated into the polystyrene film. | 606 | ~ 4% KCl rejection (diffusion experiment) | Dead-end, 1 bar | 0.785 cm^2^ (T) |  | [S10] |
|  | Double-walled CNTs were vertically grown and embedded in silicon nitride. The substrate was etched by XeF_2_. Silicon nitride was etched by ion milling. CNTs were uncapped by reactive ion etching. | 284 | Transport 1.3 nm Ru^2+^(bipyr)_3_ species but block 2 nm Au particles | Dead-end, 0.83 bar | 0.175 mm^2^ (F and T) |  | [S11] |
|  | Vertically aligned CNTs as synthesized by CVD. The substrate was removed by HF etching so that the CNTs could be compressed. The compressed CNTs were coated with epoxy resin. The two ends of the nanotubes in the membrane were opened by hand-cutting with a knife. | 2309 | Transport hexane, water, and dodecane | Gravity-driven filtration | 4 cm^2^ (F); 0.72 cm^2^ (T) | The epoxy matrix absorbed solvents such as dimethylformamide and ethanol. | [S12] |
|  | Vertically aligned CNTs were filled up with PDMS. The substrate was removed by mechanical peeling, and the membrane was sliced into thinner films. | 1203 | > 96.5% NaCl rejection | Stirred dead-end, 10 g L^-1^ NaCl, 2 bar, 20 ℃ | 1 cm^2^ (F) |  | [S13] |
|  | Vertically aligned CNTs synthesized by CVD on SiO_2_/Si substrate were filled up with polystyrene. HCl was used to remove the catalyst and delaminate the film from its substrate. Plasma treatment was used to open and functionalize the CNT tips with carboxylic and hydroxyl groups. The carboxylic groups were activated and reacted with amino groups in glycine. | ~ 650 | 87-99% NaCl rejection | Cross-flow (3 mL min^-1^), 25-27 ℃, 2.5 bar, 0.5-5 g L^-1^ NaCl | 1 cm^2^ (F) | Withstand 10 bar | [S14] |
|  | SWCNTs were electrophoretically aligned and deposited on the positive electrode. |  | ~ 60% KCl rejection | Dead-end, 10 kPa, 10 mM KCl |  |  | [S15] |
| **Zeolite film** | α-alumina supported MFI-type zeolite membranes were synthesized by the *in-situ* crystallization method (a single hydrothermal treatment). | 0.0077 | 0.013 | Cross-flow (0.2 mL min^-1^), 5.8 g L^-1^ NaCl, 25 ℃, 21 bar |  |  | [S16] |
|  | α-alumina supported MFI-type zeolite membranes were synthesized through *in-situ* crystallization. The synthesis process was repeated one more time to improve membrane density or eliminate intracrystalline pores. | 0.0065 | 0.19 | Cross-flow (0.5 mL min^-1^), 5.8 g L^-1^ NaCl, 20.7 bar | 2.5 cm^2^ (T) |  | [S17] |
|  |  | 0.015 | 7.23 | Cross-flow, 5.8 g L^-1^ NaCl, 27.6 bar | ~ 11.0 cm^2^ (F and T) | 80-hour operation stability. | [S18] |
|  |  | 0.014 | 1.86 | Cross-flow (1.5 mL min^-1^), 0.58 g L^-1^ NaCl, 20.7 bar | 25.12 cm^2^ (F) |  | [S19] |
|  | MFI-type zeolite membranes were synthesized by seeding and secondary growth on α-alumina substrates. | 0.049 | 0.57 | Cross-flow (1.0 mL min^-1^), 5.8 g L^-1^ NaCl, 27.6 bar |  |  | [S20] |
|  | Aluminum-doped ZSM-5 zeolite membranes were synthesized by seeding and secondary growth on the inner surface of tubular α-alumina substrates. | 0.21 | 1.65 | Cross-flow, 5.8 g L^-1^ NaCl, 27.6 bar | 17.6 cm^2^ (F); 11 cm^2^ (T) | 16-hour operation stability. The membrane can be regenerated easily (toluene as an organic foulant). | [S21] |
| **MOF film** | UiO-66 polycrystalline membranes were fabricated on alumina hollow fibers using an *in-situ* solvothermal synthesis method. | 0.14 | < 50% Na^+^ rejection | Dead-end, 10.0 bar, 2 g L^-1^ NaCl, 20 ± 2 ℃ | 4.0 cm^2^ (F) | Excellent stability in deionized water and various saline water; 170-h operation stability | [S22] |
|  | ZIF-8 membrane was prepared by secondary seeded growth. |  | 6% NaCl rejection with a flux of 600 kg m^-2^ h^-1^ | Cross-flow, 1.5 bar, 3 g L^-1^ NaCl | ~ 5.1 cm^2^ (F) | ZIF-8 membrane was structurally robust but interacted with seawater ions. | [S23] |
|  | Uio-66 membrane was synthesized by seed-assisted solvothermal synthesis method on the ceramic tube. |  | 49.8% Na^+^ rejection with a permeance of 0.34 L m^-2^ h^-1^ bar^-1^ | Dead-end, 10 bar, 1 g L^-1^ NaCl, 26 ℃ |  | 180-h operation stability | [S24] |
|  | Aluminum MOF-303 membranes were prepared on α-Al_2_O_3_ substrates via an *in-situ* hydrothermal synthesis method. | 0.74 | 33.2% NaCl rejection | Dead-end, 5 bar, 1 g L^-1^ NaCl, 25 ± 2 ℃ |  | Stable in saline water; 60-h operation stability | [S25] |
|  | ZIF-8 film was *in-situ* grown on a tannic acid-coated PES substrate. | 3.6 | 64.7% NaCl rejection | Cross-flow, 5 bar, 2 g L^-1^ NaCl, 25 ℃ | 36 cm^2^ (F) | 100-h operation stability and recycle stability | [S26] |
|  | TA-Zn^2+^ was prepared onto substrate by self-assembly and partially self-converted to facilitate the formation of ZIF-8 top layer. | 5.1 | 55.2% NaCl rejection | Cross-flow, 5 bar, 2 g L^-1^ NaCl | 25 cm^2^ (T); 36 cm^2^ (F) | 100-h operation stability | [S27] |
| **COF film** | COF membrane was fabricated at a water-water interface and transferred onto PAN substrates or non-woven fabric substrates. | 4.57 | 4.81 | Cross-flow, 1 g L^-1^ NaCl, 4 bar |  | 6-day operation stability | [S28] |
|  | The 3-amino-propyltriethoxysilane modified α-alumina tubes were first seeded with hydroxyl-functionalized COF crystals, and secondary grew in the COF mother solution. The formed COF membrane was modified by succinic anhydride to fabricate a carboxyl-functionalized COF membrane. | 1.65 | 7.24 | Cross-flow, 2 g L^-1^ NaCl, 2 bar | 22.6 cm^2^ (T) | Seven-day operation stability; Stable in acid (pH = 1) and alkaline (pH = 13) solutions. | [S29] |
|  | TpHz was grown on a PEI-modified PES substrate using a counter-diffusion method with two steps of growth. |  | 6.7% NaCl rejection with a permeance of ~ 100 L m^-2^ h^-1^ MPa^-1^ | Stirred dead-end, 1 g L^-1^ NaCl, 4 bar | 2.8 cm^2^ (F) |  | [S30] |
|  | Dopamine-modified PAN was used as substrate. The first layer of COF was *in-situ* grown on the substrate for 72 hours, and the secondary layer of COF was synthesized by a counter-diffusion method. | 13.1 | 45.3% NaCl rejection | Cross-flow (45 L h^-1^), 5 bar, 1 g L^-1^ NaCl | 3.14 cm^2^ (T); ~ 7 cm^2^ (F) | 168-h operation stability; The water permeance and Na_2_SO_4_ rejection did not change at 10 bar. | [S31] |
|  | The bi-layered COF nanofilms were synthesized using a two-step solvothermal synthesis strategy. |  | 95.7% Na_2_SO_4_ rejection with a flux of 1.7 L m^-2^ h^-1^; ~ 40% NaCl rejection | Stirred dead-end (500 rpm), 3 bar, 1 g L^-1^ NaCl or Na_2_SO_4_ | > 4 cm^2^ (F) | Good mechanical strength (bending) | [S32] |
|  | COF crystals were *in-situ* grown on a polyimide substrate with dopamine polymerization. Dopamine soldered the COF crystals. |  | 99.5% Na_2_SO_4_ rejection with a permeance of 51.3 L m^-2^ h^-1^ bar^-1^; 49.2% NaCl rejection | Stirred dead-end, 1 g L^-1^ NaCl or Na_2_SO_4_, 5 bar | 21.2 cm^2^ (T) | Excellent antifouling property; 100-h operation stability; Enhanced mechanical strength and thermal stability compared to pristine COF | [S33] |
|  | TpPa membrane was synthesized by counter-diffusion with acetonitrile added in the aqueous solution. A hydrolyzed PAN membrane was used as the substrate. |  | ~ 18% NaCl rejection with a permeance of ~ 10.5 L m^-2^ h^-1^ bar^-1^ | 1 bar, 1 g L^-1^ NaCl | 5.7 cm^2^ (F) | Withstand 5 bar; 140-h operation stability | [S34] |
| **Nanoporous graphene** | Single-walled CNTs and graphene were synthesized by atmospheric-pressure CVD and low-pressure CVD, respectively. Graphene was transported onto the freestanding CNT networks. A meso-SiO_2_ template was formed on the graphene surface, and an O_2_ plasma process was applied to remove the graphene located in the SiO_2_ to create pores in graphene. Finally, SiO_2_ was removed by HF vapor to make nanoporous graphene. | 110.6 | 187.9 | Cross-flow (0.5 cm s^-1^), 2 g L^-1^ NaCl, 5 bar | 18 cm^2^ (F); 1.2 cm^2^ (T) | Excellent mechanical performance (withstand ~8-10 MPa and bending); Excellent anti-biofouling property; pH stable (stable salt rejection at pH 3 after 24-hour osmotic operation) | [S35] |
|  | Single-layer graphene synthesized by CVD was transferred onto a silicon nitride microchip with a 5-μm-diameter hole. Nanopores were created in the suspended graphene layer by an oxygen plasma etching process. | 2.25 × 10^7^ | ~ 100% KCl rejection (> 98.8% KCl rejection) | Gravity-driven filtration (0.16 bar), 40 ℃ | 19.6 um^2^ (T) | Plasma-treated graphene has much weaker mechanical strength than pristine graphene. The nanoporous graphene surface might be contaminated by polymethyl methacrylate residues and airborne contaminants. | [S36] |
|  | Reactive ion etching of oxygen was used to create pores in graphene. The nanoporous graphene was supported by silicon wafers with hole arrays as a mobile membrane to limit the defects in the graphene. |  | 58-100% NaCl rejection with a permeance of 4.34-5.90 × 10^7^ L m^-2^ h^-1^ bar^-1^ | Gravity-driven filtration (~ 0.1 bar), 0.58 g L^-1^ NaCl | 1.44 mm^2^ (F) |  | [S37] |
| **Stacked GO** | GO nanosheets were crosslinked by toluidine blue O. | 0.0000000555 | 81% NaCl rejection | Dead-end (330 rpm), 0.01 M NaCl, 298 K | 63.6 cm^2^ (F); 17.3 cm^2^ (T) | 7-day operation stability. | [S38] |
|  | GO dispersions prepared by a Hummers' method were mixed with graphene dispersions prepared by the electrochemical exfoliation method. The mixture was assembled as membranes with a forward-pressure system and post-processed by thermal-pressure reduction. |  | 51.2% NaCl rejection | Dead-end, 0.1 mM NaCl |  | 24-h operation stability; stable in hypochlorous acid treatment; stable after 1-h ultrasonic vibration. | [S39] |
|  | GO was prepared by the modified Tours method. PEI-modified GO membrane was made by pressure-assembly stacking technique. | 14.125 | 20% NaCl rejection | Dead-end, 8.0 bar, 20 mM NaCl | 40 cm^2^ (T) | 12 h operation stability | [S40] |
|  | PEI-modified GO and polyacrylic acid were sequentially assembled onto a hydrolyzed PAN membrane. The nanohybrid membrane was immersed in a PVA solution and cross-linked by glutaraldehyde. |  | ((PEI-modified GO)/PAA)_1_/PVA/GA: 37.8 ± 0.2% NaCl rejection with a permeance of 12.4 ± 0.4 kg m^-2^ h^-1^ MPa^-1^ | Cross-flow, 5 bar | 21 cm^2^ (T) | Improved mechanical stability and thermal stability | [S41] |
|  |  |  | ((PEI-modified GO)/PAA)_5_/PVA/GA  43.2 ± 0.2% NaCl rejection with a permeance of 8.1 ± 0.2 kg m^-2^ h^-1^ MPa^-1^ |  |  |  |  |
|  | GO was prepared from graphite with Hummers' method. PDA-coated PSF substrate was soaked in 1,3,5-benzenetricarbonyl trichloride and GO solution alternatively to form a stacked GO membrane. | 8 - 27.6 | 6 - 19% NaCl rejection | Stirred dead-end, 3.4 bar, 1.2 g L^-1^ NaCl |  |  | [S42] |
|  | GO was prepared with a modified Hummers' method and reduced by base-refluxing (Wilson's method). GO was vacuum-filtrated onto a microfiltration substrate. | 21.8 |  | Dead-end, 5 bar, 1.2 g L^-1^ NaCl | 2.83 cm^2^ (T); ~12.8 cm^2^ (F) |  | [S43] |
|  |  | 3.3 | ~ 40% NaCl rejection |  |  |  |  |
|  | GO was prepared by a modified Hummers' method. PAN and GO nanosheets were alternatively deposited on hydrolyzed PAN support to prepare a stacked GO membrane. | 1 - 3 | ~ 15-50% NaCl rejection | Cross-flow (0.4 L min^-1^), 1.2-5.8 g L^-1^ NaCl, 20.7 bar, 25 ± 0.5 ℃ | 42 cm^2^ (T) |  | [S44] |
|  | A mixed solution of chitosan and GO nanosheets was vacuum-filtrated onto the PAN substrate to form a c-GO/PAN membrane. |  | 51.8% Na_2_SO_4_ rejection with a flux of 29.1 L m^-2^ h^-1^ | Cross-flow, 1.0 g L^-1^ Na_2_SO_4_, 8 bar, 25 ℃ | 2.9 cm^2^ (T) | Antifouling properties (against dyes); Good stability (ultrasonication in water) | [S45] |
| **Stacked nanoporous GO** | Nanopores on GO nanosheets were created by H_2_O_2_ oxidation. Nanoporous GO nanosheets were vacuum filtrated onto a PES substrate and thermally reduced. |  | ~ 40% NaCl rejection with a permeance of 39.93 L m^-2^ h^-1^ bar^-1^ | Dead-end, 6 bar, 1.2 g L^-1^ NaCl | 8.55 cm^2^ (T) | 30-day operation stability | [S46] |
|  | Holey-graphene (hGO) was synthesized by removing defective carbons from pristine graphene sheets via oxidation and a modified Hummers' method. The hGO dispersions were filtrated onto polycarbonate support to form the hGO membrane. |  | < 90% NaCl rejection with a permeance of ~ 8-80 L m^-2^ h^-1^ bar^-1^ | Dead-end, 2.7-4.8 bar, 10 g L^-1^ NaCl | 17.3 cm^2^ (F); 615.8 mm^2^ (T) |  | [S47] |
|  | GO was vacuum filtrated onto the PDA-coated cellulose acetate substrate and crosslinked by diamine or cation to form a stacked GO membrane. Nanopores in the stacked GO membrane were created by ion beam. |  | Enhanced K^+^ selectivity (mono-/di-valent metal ion selectivity) | Diffusion | 4 cm^2^ (F); 0.785 cm^2^ (T) | Excellent stability in water, acid, and alkali solutions. | [S48] |
| **Stacked MXene** | Ti_3_C_2_T_x_ nanosheets were synthesized from Ti_3_AlC_2_ and delaminated. The MXene nanosheets were deposited on the ceramic substrate and subjected to a single calcination step. | 6.91 | 0.55 | Cross-flow, 0.58 g L^-1^ NaCl, 3 bar | 27.6 cm^2^ (F) | 100-hour operation stability | [S49] |
|  | MXene nanosheets were prepared from Ti_3_AlC_2_ and delaminated via ultrasonication. MXene nanosheets were vacuum filtrated onto the PAN substrate and surface-coated with poly(ethylene imine). | ~ 8.79 | 0.51 | 2.9 g L^-1^ NaCl, 2 bar | 2.54 cm^2^ (T) |  | [S50] |
| **Stacked MoS_2_** | MoS_2_ dispersions were produced by exfoliation of MoS_2_ powder in a solvent. MoS_2_ flakes were filtrated onto polyvinylidene fluoride support and functionalized by dyes. |  | ~ 20% NaCl rejection with a permeance of ~ 270 L m^-2^ h^-1^ bar^-1^ | Dead-end, 1 bar, 58.4 g L^-1^ NaCl | ~ 0.79 cm^2^ (F) | Good mechanical property (bending); 6-month stability in water (no swelling) | [S51] |
|  | 2D MoS_2_ layers were synthesized by CVD, transferred onto a PES substrate, and sealed by PDMS with a small, opened area for filtration. | 322.7 ± 27.2 | > 99.5% NaCl rejection (diffusion, 0.6 M NaCl) | Dead-end, 1 bar | ~ 2 cm^2^ (F) |  | [S52] |
|  | MoS_2_ nanosheets were prepared by lithium intercalation and exfoliation in water. The formed single-layer MoS_2_ nanosheets were functionalized with organohalide reagents and made into membranes by vacuum filtration. |  | 82.5% NaCl rejection with a permeance of 33.7 ± 13.5 L m^-2^ h^-1^ bar^-1^ | Dead-end, 9 bar, 5.8 g L^-1^ NaCl | 9.62 cm^2^ (F); 1 cm^2^ (T) | Limited swelling in water; 15-h operation stability (dead-end, 4 bar) | [S53] |
|  | MoS_2_ nanosheets were prepared from MoS_2_ flakes by solvent-assisted liquid exfoliation and decorated with peptides. Positive peptide-decorated and negative peptide-decorated porous MoS_2_ nanosheets were mixed and reacted for 20 min, then vacuum filtrated onto Anodisc alumina. |  | 63 ± 12% NaCl rejection with a permeance of 228 L m^-2^ h^-1^ bar^-1^ | Stirred dead-end, 29.2 g L^-1^ NaCl, 1 bar | ~ 10.9 cm^2^ (F); 4.91 cm^2^ (T) | 1-month operation stability; Good chlorine resistance; Improved fouling resistance (due to the hydrophilic nature of the membrane) | [S54] |
|  | MoS_2_ nanosheets were prepared by electrochemical lithium intercalation and exfoliation. MoS_2_ nanosheets were functionalized by organohalide reagents and filtrated onto a porous polymer substrate. |  | MoS_2_-amide: 82% NaCl rejection with a permeance of 1.7 L m^-2^ h^-1^ bar^-1^ | Stirred dead-end, 1.8 g L^-1^ NaCl, 4 bar | 4.91 cm^2^ (F); 0.79 cm^2^ (T) | Limited swelling in NaCl and dye solutions | [S55] |
|  |  |  | MoS_2_-ethanol: 64% NaCl rejection with a permeance of 11.5 L m^-2^ h^-1^ bar^-1^ |  |  |  |  |

**Table S2** Research studies of TFN membranes

|  | **Fabrication method** | ***A* (L m^-2^ h^-1^ bar^-1^)** | ***A/B* (bar^-1^) or selectivity related information** | **Test conditions** | **Fabricated (F) and/ or tested (T) membrane area** | **Stability** | **Refs.** |
| --- | --- | --- | --- | --- | --- | --- | --- |
| **TiO_2_ TFN** | TiO_2_ nanoparticles were dispersed in an amine solution and incorporated into a polyamide layer through IP. | 2.94 | 5.28 | Dead-end, 2 g L^-1^ NaCl, 5 bar |  | Improved antifouling property | [S56] |
|  | Titanate nanotubes were synthesized from TiO_2_ nanoparticles by an alkaline hydrothermal method and surface modified with amino groups to form NH_2_-TNTs. The NH_2_-TNTs were dispersed in a TMC solution and embedded in the polyamide layer. | 2.39 | 6.43 | Cross-flow (32.7 cm s^-1^), 1.2 g L^-1^ NaCl, 2.5 bar | 14.62 cm^2^ (T) |  | [S57] |
|  | TiO_2_ nanoparticles were dispersed in an MPD solution and incorporated into a polyamide layer through IP. | 3.05 | 40.47 | Cross-flow (1 gallon min^-1^), 2 g L^-1^ NaCl, 15.5 bar | 348.39 cm^2^ (F) |  | [S58] |
|  | TiO_2_ nanoparticles were dispersed in a TMC solution and incorporated into a polyamide layer through IP. | 3.01 | 66.31 |  |  |  |  |
|  | TiO_2_ nanoparticles were dispersed in TMC solution and incorporated into the polyamide layer through IP | 3.66 | 2.59 | Cross-flow, 2 g L^-1^ NaCl, 20.7 bar |  | Enhanced organic fouling resistance; Robust antibacterial efficiency | [S59] |
| **Ag TFN** | MPD solution containing AgNO_3_ enabled the simultaneous *in-situ* formation of Ag nanoparticles within the polyamide layer. | 1.9 ± 0.1 | 99.3 ± 0.2% NaCl rejection | Cross-flow (1 L min^-1^), 2000 mg L^-1^ NaCl, 15.5 bar, 25 ℃ | 100 cm^2^ (F); 14.5 cm^2^ (T) | Enhanced antibacterial and antibiofouling properties. | [S60] |
|  | Ag/HPS were added into an aqueous solution and incorporated into the polyamide layer. | 4.9 | 6.76 | Cross-flow, 2 g L^-1^ NaCl, 16 bar, 25 ℃ | 24 cm^2^ (T) | 7-day operation stability; enhanced antibacterial and antifouling properties. | [S61] |
|  | Ag nanoparticles formed on the PSF substrate by a reduction reaction. The Ag-modified PSF substrate went through an IP reaction. | 2.50 ± 0.21 | 2.8 | Cross-flow, 2 g L^-1^ NaCl, 20 bar |  | Enhanced antifouling property | [S62] |
| **Silica TFN** | The silica nanoparticles were *in-situ* generated by the hydrolysis and condensation of SiCl_4_ in the organic solution and got incorporated into the polyamide matrix. | 9.7 | 9.97 | Cross-flow, 25 ℃, 2 g L^-1^ NaCl, 5-30 bar | 19.6 cm^2^ (T) |  | [S63] |
|  | Functionalized silica nanoparticles were dispersed in an MPD solution and incorporated into a polyamide matrix. | 1.15 | 1.17 | Cross-flow (4 L min^-1^), 2 g L^-1^ NaCl, 25 ± 1 ℃, 15 bar | 42 cm^2^ (T) |  | [S64] |
|  | Silica nanoparticles were added to the MPD solution and incorporated into the polyamide matrix. | 0.99 | 1.1 | 2 g L^-1^ NaCl, 17.2 bar | 18.1 cm^2^ (T) | Enhanced thermal stability | [S65] |
|  | Dendritic mesoporous silica nanoparticles were added to the MPD solution and incorporated into the polyamide layer. | 4.17 | ~ 6 | 2 g L^-1^ NaCl, 25 ℃, 16 bar | 60 cm^2^ (T) | 120-h operation stability; Withstand 50 bar and 45 ℃; Enhanced anti-fouling property. | [S66] |
|  | Tetramethoxysilane was added to the organic phase to *in-situ* form silica nanoparticles within the polyamide layer. | 4.42 | 8.91 | Cross-flow, 16 bar, 25 ℃, 2 g L^-1^ NaCl | 19.6 cm^2^ (T) | Enhanced antifouling property; 48-h operation stability. | [S67] |
| **GO TFN** | GO was prepared by Hummers' method. TiO_2_ was prepared by a hydrothermal method in which GO was partially reduced. rGO/TiO_2_ nanocomposite was dispersed into an MPD solution and embedded in a polyamide layer. | 4.14 | 24.4 | Cross-flow, 2 g L^-1^ NaCl, 15 bar | 36 cm^2^ (T) | Enhanced antifouling property and chlorine resistance | [S68] |
|  | GO, prepared by a modified Hummers' method, was dispersed in an MPD solution and then embedded in a polyamide matrix. | 2.29 | 5.09 | Cross-flow, 15 bar, 2 g L^-1^ NaCl, 22 ℃ | 14.75 cm^2^ (T) | Improved antifouling property; Improved chlorine resistance; Withstand 35 bar; Stable at pH 2-12. | [S69] |
|  | GOQD/MPD suspension was deposited onto the PSF substrate by N_2_ pressure assistance and then reacted with TMC solution to form the polyamide layer. | 2.73 | 8.72 | Cross-flow (17 cm s^-1^), 2 g L^-1^ NaCl, 16 bar | 19.6 cm^2^ (T) | 120-h operation stability; Enhanced fouling and chlorine resistance; Enhanced thermal stability. | [S70] |
|  | GOQD/AP nanocomposite was synthesized by a facile electrostatically driven method. The GOQD/AP nanocomposite was added to the MPD solution and incorporated into the polyamide layer. | 2.89 | 6.67 | Cross-flow (0.44 m s^-1^), 16 bar, 2 g L^-1^ NaCl | 18.75 cm^2^ (T) | 36-h operation stability; Strong antibacterial property (due to silver phosphate, TFN-GOQD did not show good antibacterial property); Enhanced antifouling property; Improved thermal stability. | [S71] |
|  | GO was synthesized by modified Hummers' method. GO-ZnO nanocomposite was dispersed in an MPD solution for membrane fabrication. | 1.75 | 1.99 | Cross-flow, 25 ℃, 20 bar, 2 g L^-1^ NaCl | 42 cm^2^ (T) | Enhanced chlorine resistance; Improved anti-fouling performance. | [S72] |
|  | GO, prepared by a modified Hummers' method, was dispersed in an MPD solution and incorporated into the polyamide layer. | 1.78 | 6.34 | Cross-flow, 20 bar, 2 g L^-1^ NaCl | 10 cm^2^ (T) | Enhanced chlorine resistance; Improved antibacterial property and anti-biofouling property. | [S73] |
|  | Functional GO nanosheets were formed by binding octadecylamine (ODA) with oxygen-containing groups on GO. ODA@GO nanosheets were added to the TMC solution to form the membrane. | 2.99 | 26.46 | Cross-flow (0.31 m s^-1^), 32 g L^-1^ NaCl, 55 bar, 25 ℃ | 19.63 cm^2^ (T) |  | [S74] |
|  | GO was fabricated by the Staudenmaier method and added to the TMC solution to form the membrane. | 2.3 | 1.15 | Cross-flow (8 cm s^-1^), 20 ℃, 2 g L^-1^ NaCl, 20.7 bar | 14.6 cm^2^ (T); 290 cm^2^ (F) | Improved antimicrobial and antifouling (biofouling) properties. | [S75] |
| **AQP TFN** | AQPZ-incorporated lipid vesicles were added into the MPD solution and then embedded in a polyamide layer on hollow fiber through IP. | 8 | 11.6 | Cross-flow, 0.5 g L^-1^ NaCl, 5 bar, 23 ℃ | 34.2 cm^2^ (F and T) | Withstand 5 bar; Membrane maintained its water permeability and salt rejection after long-term fouling and four cycles of chemical cleaning with strong ionic surfactant. | [S76] |
|  | MPD solution with proteoliposomes reacted with TMC solution to form the polyamide layer. | 10.92 | 13.6 | Cross-flow (0.3 m s^-1^), 2 bar, 0.5 g L^-1^ NaCl | 37.68 cm^2^ (F) | Five-month operation stability | [S77] |
|  |  | 3.14 | ~8.1 | Cross-flow (10 cm s^-1^), 50 bar, 3.5 g L^-1^ NaCl, 25 ℃ | 42 cm^2^ (T) | Triton X-100 soaking experiment resulted in reduced water permeance of membranes (NaCl rejection remained unchanged). Running/soaking the membrane at pH 3 ruptured the vesicle or disabled the water channel function of embedded AQPs. | [S78] |
|  |  | 1.79 | 6.34 | Cross-flow (~ 10 cm s^-1^), 5 bar, 0.5 g L^-1^ NaCl, 25 ℃ | 35 cm^2^ (F) |  | [S79] |
|  |  | 4.13 | 3.53 | Cross-flow (~ 10 cm s^-1^), 0.58 g L^-1^ NaCl, 10 bar, 25 ± 1 ℃ | 42 cm^2^ (T) | Good chemical stability (after soaking in three cleaning agents, ethylenediaminetetraacetic acid, NaOH, and citric acid). Sensitive to temperature and pressure. Withstand 65 ℃. Long-term stability (100-day operation, real RO feed water from water reclamation process as feed, periodically cleaned): the recovery of water permeability was higher than 90%. | [S80] |
|  |  | 1.61 | 6.3 | Cross-flow, 55 bar, 2 g L^-1^ NaCl | 300 cm^2^ (F); 42 cm^2^ (T) | Seven-day desalination stability (with seawater effluent as feed); The immobilization of proteoliposomes negligibly affected the mechanical strength of the overall polyamide layer. | [S81] |
|  |  | 0.87 | 5.1 | Cross-flow, 55 bar, 32 g L^-1^ NaCl |  |  |  |
|  | AQPZ was stabilized with an amphiphilic peptide, added to the MPD solution, and incorporated into the polyamide layer. | 0.95 | 2.3 | Cross-flow, 1 g L^-1^ NaCl, 13.8 bar, 25 ℃ | 42 cm^2^ (T) |  | [S82] |
| **AWC TFN** | I-quartet AWCs aggregates were dispersed onto PSF support and incorporated into polyamide. | 3.47 ± 0.89 | 20.41 | Cross-flow (0.9 m s^-1^), 27 ± 1 ℃, 18 bar, 5.8 g L^-1^ NaCl | 22 cm^2^ (T) | The membrane exhibited good mechanical and chemical stability in sodium dodecyl sulfate, ethanol, and citric acid solution. | [S83] |
|  |  | 2.51 ± 0.21 | 22.82 | Cross-flow (0.9 m s^-1^), 27 ± 1 ℃, 65 bar, 35 g L^-1^ NaCl |  |  |  |
|  |  | 6.9 | 35 | Cross-flow (0.9 m s^-1^), 25 ± 0.5 ℃, 15.5 bar, 5.8 g L^-1^ NaCl | 28.9 cm^2^ (T) | The membrane had better fouling resistance than the XLE membrane. | [S84] |
| **CNT TFN** | Carboxylated-SWCNT was inserted in liposomes and added to the MPD solution to form a polyamide layer. | 3.17 | 4.6 | Cross-flow, 16 bar, 2 g L^-1^ NaCl | 24 cm^2^ (T) | Enhanced fouling resistance; 48-hour operation stability | [S85] |
|  | CNT porins were added to the MPD solution and incorporated into the polyamide layer. | 5.08 | 17.8 | Cross-flow, 2 g L^-1^ NaCl, 16 bar | 24 cm^2^ (T) | Enhanced chlorine resistance. | [S86] |
|  | Functionalized CNTs were vacuum-filtrated onto the substrate and incorporated into a polyamide layer. | 1.71 | 3.73 | Cross-flow, 2 g L^-1^ NaCl, 25-27 ℃, 15.5 bar | 40 cm^2^ (F) |  | [S87] |
|  | Functionalized CNTs were dispersed in an MPD solution and embedded in polyamide via IP. | 3.98 | 8.50 | Cross-flow (2 L min^-1^), 15.5 bar, 25 ℃, 2 g L^-1^ NaCl | 42 cm^2^ (T) | Withstand 500 psi | [S88] |
|  |  | 3.73 | 11.25 | Cross-flow, 2 g L^-1^ NaCl, 25 ℃, 15.5 bar |  | Enhanced fouling resistance to BSA | [S89] |
|  |  | 2.23 | 3.86 | Cross-flow, 15 bar, 2 g L^-1^ NaCl | 36 cm^2^ (T) | Enhanced antifouling properties | [S90] |
|  |  | 3.31 | 2.49 | Cross-flow (11.8 cm s^-1^), 25 ℃, 15.5 bar, 2 g L^-1^ NaCl | 400 cm^2^ (F); 22.44 cm^2^ (T) | Slightly enhanced biofouling resistance | [S91] |
| **Zeolite TFN** | Zeolite nanoparticles were dispersed in an organic solution and embedded into a polyamide layer. | 3.46 | 1.07 | Stirred dead-end, 2 g L^-1^ NaCl, 15.5 bar | 13.8 cm^2^ (T) |  | [S92] |
|  |  | 2.08 | 12.47 | Cross-flow, 55.2 bar, 32 g L^-1^ NaCl | 19.4 cm^2^ (T) |  | [S93] |
|  |  | 1.22 | 0.89 | Cross-flow, 12.1 bar, 2 g L^-1^ NaCl, 25 ℃ | 35 cm^2^ (T) | Enhanced thermal stability | [S94] |
|  |  | 2.99 | 7.24 | Cross-flow (90 L h^-1^), 2 g L^-1^ NaCl, 16 bar, 25 ℃ | 144 cm^2^ (F); 38.5 cm^2^ (T) |  | [S95] |
|  |  | 2.57 | 1.64 | Cross-flow (~ 20 cm s^-1^), 2.5 bar, 0.5 g L^-1^ NaCl, 20 ± 0.5 ℃ | 42 cm^2^ (T) |  | [S96] |
|  |  | 5.11 | 4.00 | Cross-flow, 2 g L^-1^ NaCl, 16 bar, 25 ℃ |  | Stability in pH = 5 acid and multivalent cation solutions | [S97] |
|  |  | 2.46 | 3.96 | 2 g L^-1^ NaCl, 16 bar, 25 ℃ | 44 cm^2^ (T) |  | [S98] |
|  | TFN: Zeolite-A nanoparticles were dispersed in a TMC solution and incorporated into the polyamide layer via IP. | ~ 1.95 | ~ 1.35 | Stirred dead-end, 0.58 g L^-1^ NaCl, 15.5 bar | 13.85 cm^2^ (T) | Improved resistance to physical compaction | [S99] |
|  | nTFN: Zeolite-A nanoparticles were incorporated into the PES substrate and polyamide layer. | ~ 4.57 | ~ 1.99 |  |  |  |  |
|  | Zeolite nanoparticles were dispersed in an MPD solution and incorporated into a polyamide layer via IP. | 7.25 | 12.26 | Cross-flow, 15.5 bar, 25 ℃, 2 g L^-1^ NaCl | 113.8 cm^2^ (F); 28.6 cm^2^ (T) |  | [S100] |
|  | Zeolite nanoparticles were dispersed in an MPD solution and incorporated into a polyamide layer via IP. | 3.53 | 3.79 | Cross-flow (2.5 L min^-1^), 20.7 bar, 1.5 g L^-1^ NaCl | 14.62 cm^2^ (T) |  | [S101] |
| **MOF TFN** | MOF nanoparticles were dispersed in a TMC solution and incorporated into a polyamide layer via IP. | 4.02 | 8.55 | Cross-flow (0.37 m s^-1^), 15.5 bar, 2 g L^-1^ NaCl, 23 ℃ | 33.5 cm^2^ (T) |  | [S102] |
|  |  | 4.99 | 9.67 | Cross-flow, 2 g L^-1^ NaCl, 20.7 bar, 25 ℃ | 17.3 cm^2^ (T) |  | [S103] |
|  |  | 3.33 | 10 | Dead-end, 2 bar, 1 g L^-1^ NaCl | 9.5 cm^2^ (T) |  | [S104] |
|  |  | 4.46 | 21.19 | Cross-flow, 2 g L^-1^ NaCl, 15.5 bar, 25 ℃ |  | Enhanced boron rejection | [S105] |
|  |  | 7.03 | 28.77 | Cross-flow, 32 g L^-1^ NaCl, 55 bar, 25 ℃ |  |  |  |
|  |  | 3.38 | 3.38 | Cross-flow (~ 22 cm s^-1^), 2 g L^-1^ NaCl, 16 bar, 24 ± 1 ℃ | 20.02 cm^2^ (T) | Water-stable; Antibacterial properties and anti-biofouling properties (which may be attributed to the copper in the MOF) | [S106] |
|  | ZIF-8 was *in-situ* grown on Noria-PEI modified PSF substrate. IP reaction was performed on top. | 4.42 | 9.70 | Cross-flow, 2 g L^-1^ NaCl, 15.5 bar, 25 ℃ | 0.014 m^2^ (F); 18.5 cm^2^ (T) | Enhanced antifouling property; Sixty-hour operation stability | [S107] |
|  | 2D-MOF nanosheets were dispersed in an MPD solution and incorporated into the polyamide layer via IP. | 8.83 | 29.75 | Cross-flow, 4 g L^-1^ NaCl, 20 bar, 25 ± 2 ℃ | 24 cm^2^ (T) | Enhanced antifouling property; 48-hour operation stability | [S108] |
|  | ZIF-8 nanocrystals are grown *in-situ* on substrates and wrapped into a polyamide layer. | 4.74 | 14.70 | Cross-flow, 15.5 bar, 25 ℃, 2 g L^-1^ NaCl | 18.5 cm^2^ (T) | Enhanced fouling resistance | [S109] |
| **COF TFN** | COF was dispersed in an MPD solution and incorporated into the polyamide layer via IP. | 3.87 | 2.13 | 10 bar | 19.5 cm^2^ (T) | Withstand 24 bar | [S110] |
|  |  | 0.50 | 6.09 | Cross-flow (35 L h^-1^), 10 bar, 25 ℃, 2 g L^-1^ NaCl | 28.3 cm^2^ (T) |  | [S111] |
|  | MPD was added to the COF suspensions and reacted with TMC to form a polyamide layer on top of the PSF substrate. |  | 97.63% NaCl rejection with a permeance of 2.2 L m^-2^ h^-1^ bar^-1^ | Cross-flow (60 L h^-1^), 1 g L^-1^ NaCl, 6 bar, 23 ± 1 ℃ | 11.34 cm^2^ (T) | Enhanced antimicrobial properties and chlorine resistance | [S112] |
| **MXene TFN** | Ti_3_C_2_T_x_, prepared from Ti_3_AlC_2_, was dispersed into an MPD solution and incorporated into a polyamide layer via IP. | 2.96 | 7.20 | Cross-flow, 2 g L^-1^ NaCl, 16 bar, 25 ℃ | 70 cm^2^ (T) | Enhanced anti-fouling and chlorine resistance | [S113] |
|  | MXene nanosheets were prepared by selective etching of the aluminum layer from Ti_3_AlC_2_ and modified with tannic acid. The nanosheets were added to the MPD solution and incorporated into the polyamide layer via IP. | 2.4 ± 0.1 | 96% NaCl rejection | Stirred dead-end, 2 g L^-1^ NaCl, 20 bar, 25 ℃ | 14.6 cm^2^ (T) | Improved fouling resistance | [S114] |
| **MoS_2_ TFN** | MoS_2_ nanosheets were prepared from MoS_2_ flakes by solvent-assisted liquid exfoliation. MoS_2_ nanosheets were dispersed in the organic phase and incorporated into the polyamide layer via IP. | 8.04 | 15.42 | Cross-flow (60 L h^-1^), 2 g L^-1^ NaCl, 15.5 bar, 25 ℃ | 247 cm^2^ (F); 42 cm^2^ (T) | Improved fouling resistance; Loss of MoS_2_ nanosheets in the retentate, poor dispersion in the polyamide matrix; Twenty-hour operation stability | [S115] |

Note: AQPZ Aquaporin-Z; TMC Trimesoyl chloride; BSA Bovine serum albumin; PES Polyethersulfone; PEI Polyetherimide; PVA Polyvinyl alcohol; PDMS Polydimethylsiloxane

**Appendix C: Rubrics for radar charts**


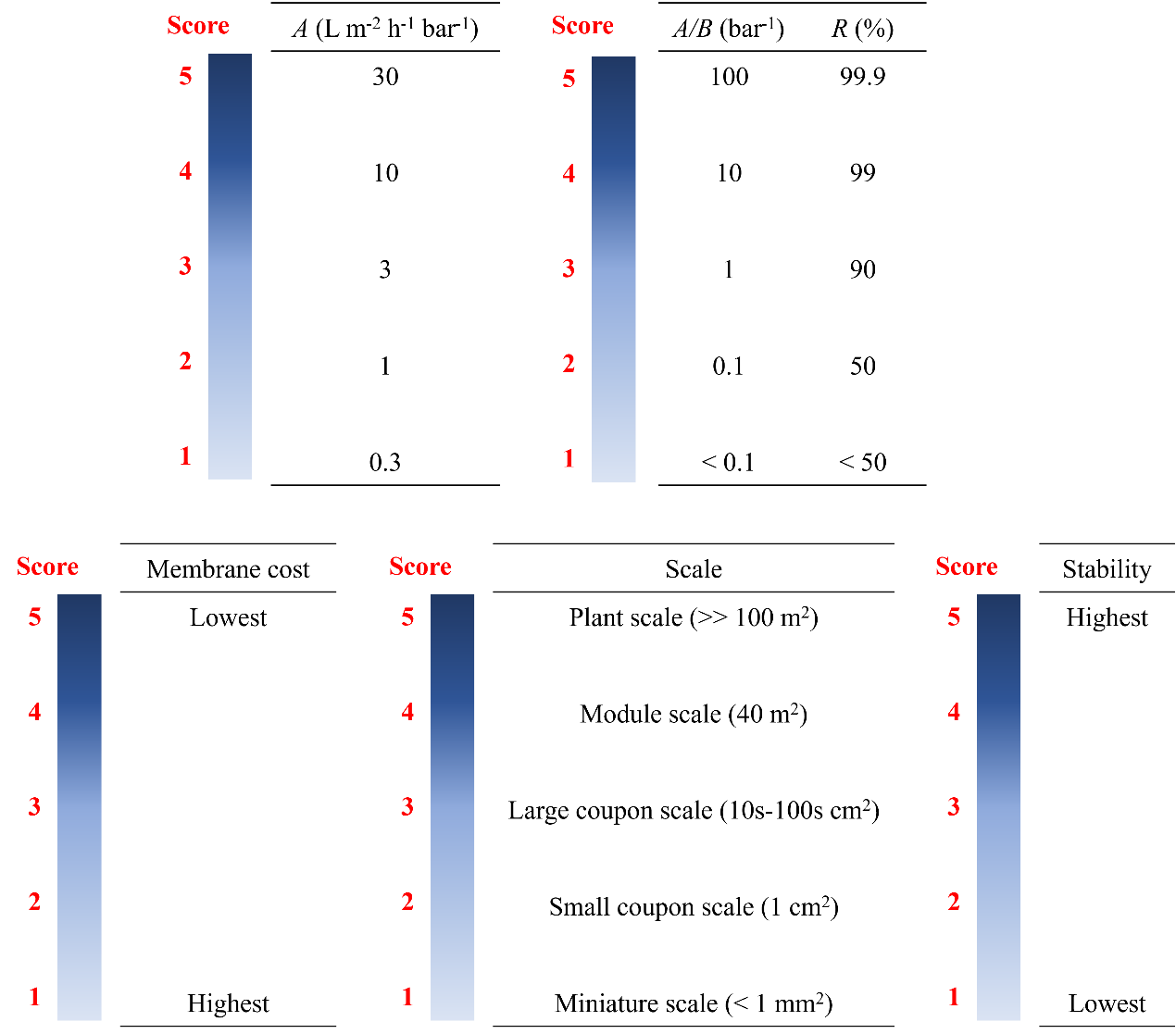


**Fig. S1** Rubrics for permeance, selectivity, membrane cost, scale, and stability

The rubrics used for developing the radar charts are shown in Fig. S1. The higher the score in each dimension, the better the membrane is. The scores of permeance and selectivity are based on *A* and *A/B* values, respectively. Conventional TFC membranes typically have a water permeance in the range of 1-5 L m^-2^ h^-1^ bar^-1^ and a selectivity in the range of 5-30 bar^-1^, which results in a score of 3 for permeance and 4 for selectivity. NaCl rejection (*R*) is used as a supplementary indicator for the selectivity score when the *A/B* value is not available. Based on the solution-diffusion model [S1, S2], *R* is related to *A/B* through the following equation:

$R=\left[ \frac{A}{B}\left( \Delta P-\Delta\pi_{b}f_{cp} \right) \right]/[\frac{A}{B}(\Delta P-\Delta\pi_{b}f_{cp})+f_{cp}]$ (*S4*)

Membrane cost can be systematically scored based on two critical considerations: the cost of materials involved and the cost of membrane fabrication. Conventional TFC polyamide membranes, scoring 5 in membrane cost, serve as a good benchmark for the evaluation of other membranes (Fig. S2). Typical TFC membranes can be routinely fabricated in large-scale roll-to-roll production without involving any costly chemicals or fabrication steps. Similar production lines can be adopted for TFN membranes, although the dispersion of nanofillers could cause additional complications. Some TFN membranes may involve expensive nanofillers but often at low dosages (e.g., 30 µg cm^-2^ for CNT [S116] and 50 µg cm^-2^ for COF [S112]), which could cause moderate increases in the material cost. Therefore, TFN membranes are still relatively competitive with respect to the overall membrane cost, leading to a high score in the range of 4 - 4.5 depending on the cost of nanofillers. In contrast, most novel G3 membranes involve either demanding fabrication processes (e.g., VA-CNT [S117] and nanoporous graphene [S35]) or expensive materials (e.g., AQP SLB [S118-S120] and nanoporous graphene [S35]), leading to less competitive scores for membrane cost. The score for scale is based on the membrane area. As a useful reference, a standard RO module (i.e., a “4080 element”) with approximately 40 m^2^ is assigned a score of 4 – representing good readiness for pilot testing and potential practical implementation Since conventional TFC membranes are produced in millions of m^2^, a score of 5 is given. Membranes with smaller areas are scored based on: 1 for miniature scale (< 1 mm^2^), 2 for small coupon scale (on the order of 1 cm^2^), and 3 for large coupon scale (10 s to 100 s cm^2^).


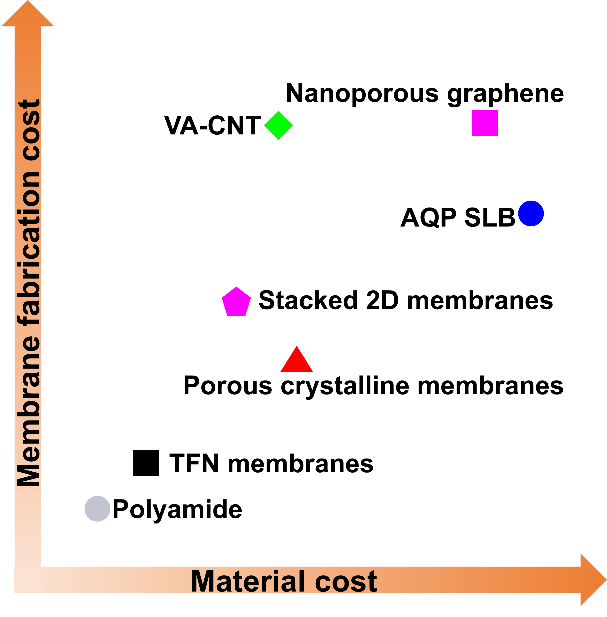


**Fig. S2** Comparison of membrane cost

The score for stability is derived from the mechanical stability, thermal stability, chemical stability, and fouling stability of membranes (Table S3). If the membrane has poor mechanical properties or chemical stability (e.g., unstable in water), the lowest score of 1 is assigned to this membrane since it can unlikely be applied for practical applications. Otherwise, the score is determined by adding a credit or imposing a penalty in comparison with the benchmark TFC membranes (see Table S3 for details, with a baseline score of 3 assigned to TFC membranes, considering their poor chlorine resistance and high fouling propensity). If the score is ≤ 2 based on basic requirements, extended requirements are not considered. Otherwise, scores are added if the membrane has special properties, such as thermal- or acid-stability. Figure 4 adopts a dark-red region to represent the current state (based on available experimental data) and a light-red region (based on theoretical estimates). For evaluating the current state with respect to stability, credits will be assigned only if experimental evidence of improved stability (e.g., chlorine/fouling resistance, Table S3) has been demonstrated. However, to evaluate the ultimate potential, credits will be applied to theoretical stability based on the fundamental properties of materials.

**Table S3** Rubrics for stability

| **Basic requirements (in comparison with conventional TFC membranes)** | | |
| --- | --- | --- |
|  | Reported properties | Actions on score |
| **Mechanical stability** | Withstand ≥ 10 bar (reasonable mechanical stability) | None |
|  | Cannot withstand 10 bar or has compromised mechanical stability | -1 |
| **Thermal stability** | Withstand ≥ 45 ℃ (reasonable thermal stability) | None |
|  | Cannot withstand 45 ℃ | -1 |
| **Chemical stability** | Enhanced chlorine resistance | +0.5 |
|  | Does not degrade under chlorine attack | +1 |
|  | Compromised chemical stability (e.g., oxidization under ambient conditions) | -1 |
| **Fouling stability** | Enhanced fouling resistance | +0.5 |
| **Extended Requirements** | | |
|  | Reported properties | Actions on score |
| **Thermal stability** | high thermal stability | +1 |
| **Chemical stability** | stable in acidic (pH ≤ 1) or basic (pH ≥ 13) solutions | +1 |

**Table S4** Stability of G3 membranes

| Membranes | Mechanical stability | Thermal stability | Chemical stability | Fouling stability |
| --- | --- | --- | --- | --- |
| AQP SLB | Operation pressure ≤ 5 bar; lipid mobility | N.A. | Potential degradation of lipid layer [S4] and protein denaturation | N.A. |
| VA-CNT | Withstand at least 10 bar [S14] | Withstand 45 ℃ [S121] | CNT tips were functionalized in hypochlorite exposure [S122]; VA-CNT membrane could be stable in acid and basic solutions. | Supposed to be anti-biofouling due to the antimicrobial properties of CNTs |
| Zeolite film | Withstand at least 10 bar [S17] | Withstand 80 ℃ [S123] | Isomorphous replacement [S124] in which Al^3+^ and Si^4+^ in zeolites are replaced by other elements; supposed to have good chlorine resistance. | N.A. |
| MOF film | Withstand at least 10 bar [S22, S24] | Withstand 50 ℃ [S22] | Stable in hypochlorite solutions [S125]. In SAPO-34, Al was partially replaced by other elements [S23]. | N.A. |
| COF film | Withstand at least 10 bar [S126] | Withstand 76 ℃ [S127]; supposed to be thermal stable | Stable in acid and basic solutions [S29, S128]; supposed to have good chlorine resistance; | N.A. |
| Nanoporous graphene | Problematic mechanical stability and lack of experience in handling such thin film | Withstand 40 ℃ [S36] | Supposed to be stable in acid and alkali aqueous solutions. | Graphene is considered an antibacterial material, but the fouling propensity of nanoporous graphene lacks deep investigation. |
| Stacked GO/ stacked nanoporous GO | Interlayer spacing can be altered by operation pressure [S45] | N.A. | Interlayer spacing can be altered by solution chemistry [S129, S44]; stable in acid and basic solutions [S48]; supposed to have good chlorine resistance as GO is oxidized. | Disputable [S130] |
| Stacked MXene | Lack of data on operation pressure ≥ 5 bar | N.A. | Interlayer spacing can be altered by solution chemistry [S131-S133]; stable in hypochlorite solutions [S134]; MXenes are easily oxidized under ambient conditions [S135]. | Good antifouling and antibacterial properties [S136] |
| Stacked MoS_2_ | Withstand at least 9 bar [S53] | N.A. | Stable in hypochlorite solutions [S54]; MoS_2_ is easily oxidized under ambient conditions [S137]. | N.A. |

**Appendix D: Separate radar charts for each TFN membrane**

**
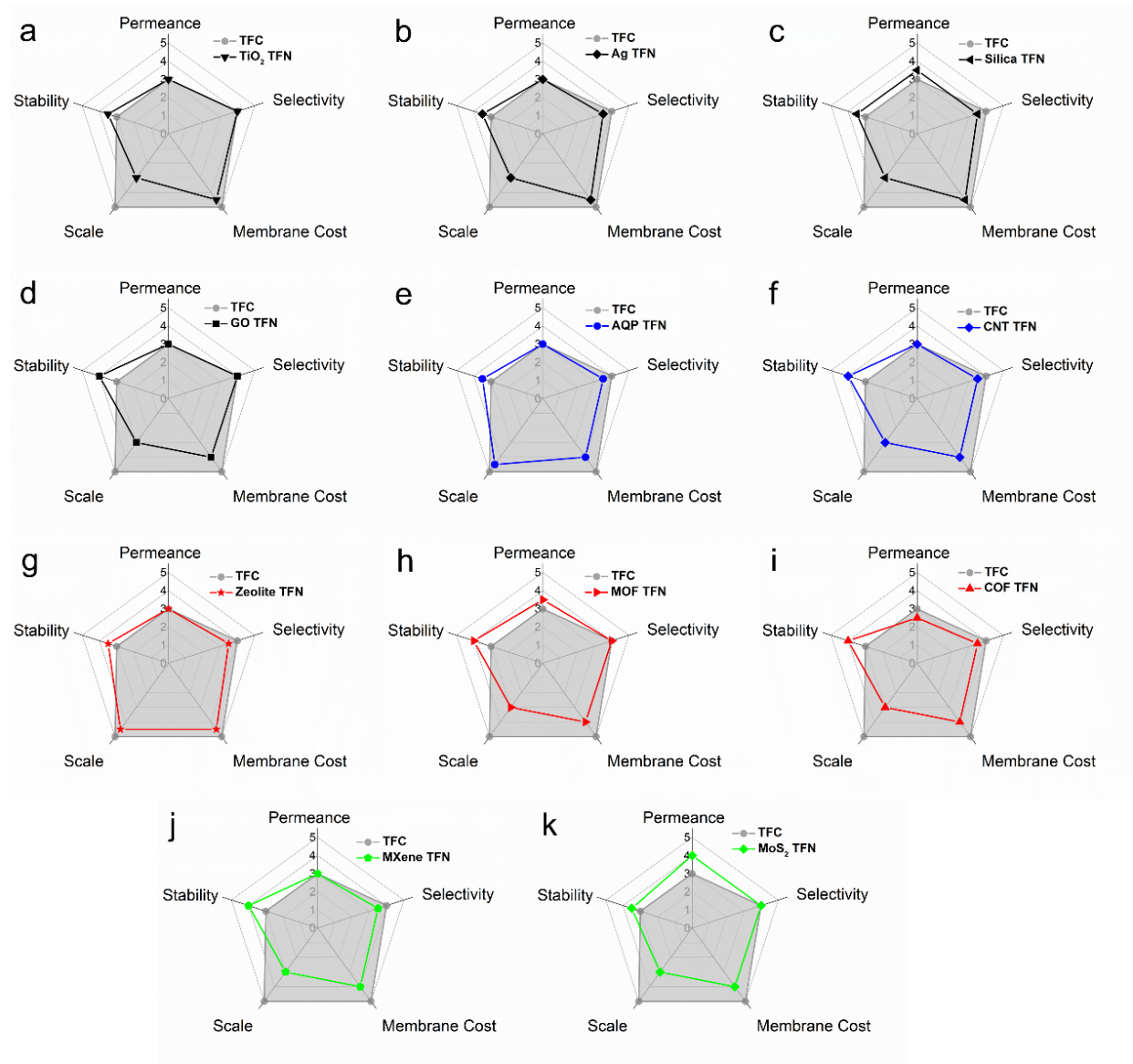
**

**Fig. S3** Separate radar charts for each TFN membrane. (**a**) TiO_2_ TFN membrane, (**b**) Ag TFN membrane, (**c**) silica TFN membrane, (**d**) GO TFN membrane, (**e**) AQP TFN membrane, (**f**) CNT TFN membrane, (**g**) zeolite TFN membrane, (**h**) MOF TFN membrane, (**i**) COF TFN membrane, (**j**) MXene TFN membrane, (**k**) MoS_2_ TFN membrane

**

**

**Fig. S4** A radar chart for TFWC membrane

**References**

[S1] D.R. Paul Reformulation of the solution-diffusion theory of reverse osmosis. J. Membr. Sci. **241**, 371–386 (2004). <https://doi.org/10.1016/j.memsci.2004.05.026>

[S2] R.W. Baker, *Membrane Technology and Applications* (Wiley, 2012). <https://doi.org/10.1002/9781118359686>

[S3] J.R. Werber, A. Deshmukh, M. Elimelech, The critical need for increased selectivity, not increased water permeability, for desalination membranes, Environ. Sci. Technol. Lett. **3**, 112–120 (2016). <https://doi.org/10.1021/acs.estlett.6b00050>

[S4] M. Wang, Z. Wang, X. Wang, S. Wang, W. Ding, C. Gao, Layer-by-layer assembly of aquaporin Z-incorporated biomimetic membranes for water purification, Environ. Sci. Technol. **49**, 3761–3768 (2015). <https://doi.org/10.1021/es5056337>

[S5] P.H.H. Duong, T.-S. Chung, K. Jeyaseelan, A. Armugam, Z. Chen et al., Planar biomimetic aquaporin-incorporated triblock copolymer membranes on porous alumina supports for nanofiltration. J. Membr. Sci. **409**, 34–43 (2012). <https://doi.org/10.1016/j.memsci.2012.03.004>

[S6] P.S. Zhong, T.-S. Chung, K. Jeyaseelan, A. Armugam, Aquaporin-embedded biomimetic membranes for nanofiltration. J. Membr. Sci. **407**, 27–33 (2012). <https://doi.org/10.1016/j.memsci.2012.03.033>

[S7] X. Li, R. Wang, F. Wicaksana, C. Tang, J. Torres et al., Preparation of high performance nanofiltration (NF) membranes incorporated with aquaporin Z. J. Membr. Sci. **450**, 181–188 (2014). <https://doi.org/10.1016/j.memsci.2013.09.007>

[S8] W. Ding, J. Cai, Z. Yu, Q. Wang, Z. Xu, Z. Wang, C. Gao, Fabrication of an aquaporin-based forward osmosis membrane through covalent bonding of a lipid bilayer to a microporous support, J. Mater. Chem. A **3**, 20118–20126 (2015). <https://doi.org/10.1039/c5ta05751e>

[S9] B.J. Hinds, N. Chopra, T. Rantell, R. Andrews, V. Gavalas, L.G. Bachas, Aligned multiwalled carbon nanotube membranes, Science **303**, 62–65 (2004). <https://doi.org/10.1126/science.1092048>

[S10] M. Majumder, N. Chopra, R. Andrews, B.J. Hinds, Enhanced flow in carbon nanotubes, Nature **438**, 44 (2005). <https://doi.org/10.1038/43843a>

[S11] J.K. Holt, H.G. Park, Y. Wang, M. Stadermann, A.B. Artyukhin, C.P. Grigoropoulos, A. Noy, O. Bakajin, Fast mass transport through sub–2-nanometer carbon nanotubes, science **312**, 1034–1037 (2006). <https://doi.org/10.1126/science.1126298>

[S12] F. Du, L. Qu, Z. Xia, L. Feng, L. Dai, Membranes of vertically aligned superlong carbon nanotubes, Langmuir **27**, 8437–8443 (2011). <https://doi.org/10.1021/la200995r>

[S13] S. Trivedi, K. Alameh, Effect of vertically aligned carbon nanotube density on the water flux and salt rejection in desalination membranes. SpringerPlus **5**, 1158 (2016). <https://doi.org/10.1186/s40064-016-2783-3>

[S14] S. Vahdatifar, A. Ali Khodadadi, Y. Mortazavi, L.F. Greenlee, Functionalized open-ended vertically aligned carbon nanotube composite membranes with high salt rejection and enhanced slip flow for desalination. Sep. Purif. Technol. **279**, 119773 (2021). <https://doi.org/10.1016/j.seppur.2021.119773>

[S15] D.C. Yang, R.J. Castellano, R.P. Silvy, S.K. Lageshetty, R.F. Praino et al., Fast water transport through subnanometer diameter vertically aligned carbon nanotube membranes. Nano Lett. **23**, 4956–4964 (2023). <https://doi.org/10.1021/acs.nanolett.3c00797>

[S16] L. Li, J. Dong, T.M. Nenoff, R. Lee, Reverse osmosis of ionic aqueous solutions on a MFI zeolite membrane, Desalination **170**, 309–316 (2004). <https://doi.org/10.1016/j.desal.2004.02.102>

[S17] L. Li, J. Dong, T.M. Nenoff, R. Lee, Desalination by reverse osmosis using MFI zeolite membranes, J. Membr. Sci. **243**, 401–404 (2004). <https://doi.org/10.1016/j.memsci.2004.06.045>

[S18] N. Liu, L. Li, B. McPherson, R. Lee, Removal of organics from produced water by reverse osmosis using MFI-type zeolite membranes, J. Membr. Sci. **325**, 357–361 (2008). <https://doi.org/10.1016/j.memsci.2008.07.056>

[S19] L. Li, N. Liu, B. McPherson, R. Lee, Influence of counter ions on the reverse osmosis through MFI zeolite membranes: implications for produced water desalination, Desalination **228**, 217–225 (2008). <https://doi.org/10.1016/j.desal.2007.10.010>

[S20] L. Li, N. Liu, B. McPherson, R. Lee, Enhanced water permeation of reverse osmosis through mfi-type zeolite membranes with high aluminum contents, Ind. Eng. Chem. Res. **46**, 1584–1589 (2007). <https://doi.org/10.1021/ie0612818>

[S21] J. Lu, N. Liu, L. Li, R. Lee, Organic fouling and regeneration of zeolite membrane in wastewater treatment, Sep. Purif. Technol. **72**, 203–207 (2010). <https://doi.org/10.1016/j.seppur.2010.02.010>

[S22] X. Liu, N.K. Demir, Z. Wu, K. Li, Highly water-stable zirconium metal-organic framework UiO-66 membranes supported on alumina hollow fibers for desalination, J. Am. Chem. Soc. **137**, 6999–7002 (2015). <https://doi.org/10.1021/jacs.5b02276>

[S23] M.C. Duke, B. Zhu, C.M. Doherty, M.R. Hill, A.J. Hill, M.A. Carreon, Structural effects on SAPO-34 and ZIF-8 materials exposed to seawater solutions, and their potential as desalination membranes, Desalination **377**, 128–137 (2016). <https://doi.org/10.1016/j.desal.2015.09.004>

[S24] X. Ren, X. Zhang, D. Tang, A. Yang, Y. Feng, Decorating a metal−organic framework UiO-66 layer on ceramics substrate by the seed-assisted solvothermal method for high-performance desalination, Desalination Water Treat. **161**, 156–160 (2019). <https://doi.org/10.5004/dwt.2019.24307>

[S25] S. Cong, Y. Yuan, J. Wang, Z. Wang, F. Kapteijn, X. Liu, Highly water-permeable metal-organic framework MOF-303 membranes for desalination, J. Am. Chem. Soc. **143**, 20055–20058 (2021). <https://doi.org/10.1021/jacs.1c10192>

[S26] Y. Xu, Y. Xiao, W. Zhang, H. Lin, L. Shen et al., Plant polyphenol intermediated metal-organic framework (MOF) membranes for efficient desalination. J. Membr. Sci. **618**, 118726 (2021). <https://doi.org/10.1016/j.memsci.2020.118726>

[S27] Y. Xiao, W. Zhang, Y. Jiao, Y. Xu, H. Lin, Metal-phenolic network as precursor for fabrication of metal-organic framework (MOF) nanofiltration membrane for efficient desalination. J. Membr. Sci. **624**, 119101 (2021). <https://doi.org/10.1016/j.memsci.2021.119101>

[S28] H. Wang, J. Zhao, Y. Li, Y. Cao, Z. Zhu, M. Wang, R. Zhang, F. Pan, Z. Jiang, Aqueous two-phase interfacial assembly of COF membranes for water desalination, Nano-Micro Letters **14**, 216 (2022). <https://doi.org/10.1007/s40820-022-00968-5>

[S29] C. Liu, Y. Jiang, A. Nalaparaju, J. Jiang, A. Huang, Post-synthesis of a covalent organic framework nanofiltration membrane for highly efficient water treatment, J. Mater. Chem. A **7**, 24205–24210 (2019). <https://doi.org/10.1039/c9ta06325k>

[S30] R. Wang, M. Wei, Y. Wang, Secondary growth of covalent organic frameworks (COFs) on porous substrates for fast desalination. J. Membr. Sci. **604**, 118090 (2020). <https://doi.org/10.1016/j.memsci.2020.118090>

[S31] J. Shen, J. Yuan, B. Shi, X. You, R. Ding et al., Homointerface covalent organic framework membranes for efficient desalination. J. Mater. Chem. A **9**, 23178–23187 (2021). <https://doi.org/10.1039/D1TA06439H>

[S32] A. Xiao, X. Shi, Z. Zhang, C. Yin, S. Xiong, Y. Wang, Secondary growth of bi-layered covalent organic framework nanofilms with offset channels for desalination, J. Membr. Sci. **624**, 119122 (2021). <https://doi.org/10.1016/j.memsci.2021.119122>

[S33] Y. Zhang, J. Guo, G. Han, Y. Bai, Q. Ge et al., Molecularly soldered covalent organic frameworks for ultrafast precision sieving. Sci. Adv. **7**, eabe8706 (2021). <https://doi.org/10.1126/sciadv.abe8706>

[S34] Y. Wu, Y. Wang, F. Xu, K. Qu, L. Dai, H. Cao, Y. Xia, L. Lei, K. Huang, Z. Xu, Solvent-induced interfacial polymerization enables highly crystalline covalent organic framework membranes, J. Membr. Sci. **659**, 120799 (2022). <https://doi.org/10.1016/j.memsci.2022.120799>

[S35] Y. Yang, X. Yang, L. Liang, Y. Gao, H. Cheng, X. Li, M. Zou, A. Cao, R. Ma, Q. Yuan, X. Duan, Large-area graphene-nanomesh/ carbon-nanotube hybrid membranes for ionic and molecular nanofiltration, Science **364**, 1057–1062 (2019). <https://doi.org/10.1126/science.aau5321>

[S36] S.P. Surwade, S.N. Smirnov, I.V. Vlassiouk, R.R. Unocic, G.M. Veith, S. Dai, S.M. Mahurin, Water desalination using nanoporous single-layer graphene, Nat. Nanotechnol. **10**, 459–464 (2015). <https://doi.org/10.1038/nnano.2015.37>

[S37] A.S. Kazemi, Y. Abdi, J. Eslami, R. Das, Support based novel single layer nanoporous graphene membrane for efficacious water desalination, Desalination **451**, 148–159 (2019). <https://doi.org/10.1016/j.desal.2018.03.003>

[S38] Z. Wang, C. Ma, C. Xu, S.A. Sinquefield, M.L. Shofner, S. Nair, Graphene oxide nanofiltration membranes for desalination under realistic conditions, Nat. Sustain. **4**, 402–408 (2021). <https://doi.org/10.1038/s41893-020-00674-3>

[S39] P. Zhang, Y. Wang, P. Li, X. Luo, J. Feng et al., Improving stability and separation performance of graphene oxide/graphene nanofiltration membranes by adjusting the laminated regularity of stacking-sheets. Sci. Total Environ. **827**, 154175 (2022). <https://doi.org/10.1016/j.scitotenv.2022.154175>

[S40] Z.K. Ghouri, K. Elsaid, D.J. Hughes, M.M. Nasef, A. Abdel-Wahab et al., Strong improvement of permeability and rejection performance of graphene oxide membrane by engineered interlayer spacing. J. Membr. Sci. Lett. **3**, 100065 (2023). <https://doi.org/10.1016/j.memlet.2023.100065>

[S41] N. Wang, S. Ji, G. Zhang, J. Li, L. Wang, Self-assembly of graphene oxide and polyelectrolyte complex nanohybrid membranes for nanofiltration and pervaporation. Chem. Eng. J. **213**, 318–329 (2012). <https://doi.org/10.1016/j.cej.2012.09.080>

[S42] M. Hu, B. Mi, Enabling graphene oxide nanosheets as water separation membranes, Environ. Sci. Technol. **47**, 3715–3723 (2013). <https://doi.org/10.1021/es400571g>

[S43] Y. Han, Z. Xu, C. Gao, Ultrathin graphene nanofiltration membrane for water purification, Adv. Funct. Mater. **23**, 3693–3700 (2013). <https://doi.org/10.1002/adfm.201202601>

[S44] Y. Oh, D.L. Armstrong, C. Finnerty, S. Zheng, M. Hu, A. Torrents, B. Mi, Understanding the pH-responsive behavior of graphene oxide membrane in removing ions and organic micropollulants, J. Membr. Sci. **541**, 235–243 (2017). <https://doi.org/10.1016/j.memsci.2017.07.005>

[S45] C. Xing, J. Han, X. Pei, Y. Zhang, J. He, R. Huang, S. Li, C. Liu, C. Lai, L. Shen, A.K. Nanjundan, S. Zhang, Tunable graphene oxide nanofiltration membrane for effective dye/salt separation and desalination, ACS Appl. Mater. Interfaces **13**, 55339–55348 (2021). <https://doi.org/10.1021/acsami.1c16141>

[S46] Y. Li, W. Zhao, M. Weyland, S. Yuan, Y. Xia, H. Liu, M. Jian, J. Yang, C.D. Easton, C. Selomulya, X. Zhang, Thermally reduced nanoporous graphene oxide membrane for desalination, Environ. Sci. Technol. **53**, 8314–8323 (2019). <https://doi.org/10.1021/acs.est.9b01914>

[S47] C. Buelke, A. Alshami, J. Casler, Y. Lin, M. Hickner, I.H. Aljundi, Evaluating graphene oxide and holey graphene oxide membrane performance for water purification, J. Membr. Sci. **588**, 117195 (2019). <https://doi.org/10.1016/j.memsci.2019.117195>

[S48] Y. Wei, Z. Pastuovic, C. Shen, T. Murphy, D.B. Gore, Ion beam engineered graphene oxide membranes for mono-/ di-valent metal ions separation. Carbon **158**, 598–606 (2020). <https://doi.org/10.1016/j.carbon.2019.11.031>

[S49] Y. Sun, S. Li, Y. Zhuang, G. Liu, W. Xing, W. Jing, Adjustable interlayer spacing of ultrathin MXene-derived membranes for ion rejection, J. Membr. Sci. **591**, 117350 (2019). <https://doi.org/10.1016/j.memsci.2019.117350>

[S50] B. Meng, G. Liu, Y. Mao, F. Liang, G. Liu, W. Jin, Fabrication of surface-charged MXene membrane and its application for water desalination, J. Membr. Sci. **623**, 119076 (2021). <https://doi.org/10.1016/j.memsci.2021.119076>

[S51] W. Hirunpinyopas, E. Prestat, S.D. Worrall, S.J. Haigh, R.A.W. Dryfe, M.A. Bissett, Desalination and nanofiltration through functionalized laminar MoS_2_ membranes, ACS Nano **11**, 11082–11090 (2017). <https://doi.org/10.1021/acsnano.7b05124>

[S52] H. Li, T.J. Ko, M. Lee, H.S. Chung, S.S. Han et al., Experimental realization of few layer two-dimensional MoS_2_ membranes of near atomic thickness for high efficiency water desalination. Nano Lett. **19**, 5194–5204 (2019). <https://doi.org/10.1021/acs.nanolett.9b01577>

[S53] L. Ries, E. Petit, T. Michel, C.C. Diogo, C. Gervais, C. Salameh, M. Bechelany, S. Balme, P. Miele, N. Onofrio, D. Voiry, Enhanced sieving from exfoliated MoS_2_ membranes via covalent functionalization, Nat. Mater. **18**, 1112–1117 (2019). <https://doi.org/10.1038/s41563-019-0464-7>

[S54] B. Sapkota, W. Liang, A. VahidMohammadi, R. Karnik, A. Noy, M. Wanunu, High permeability sub-nanometre sieve composite MoS_2_ membranes, Nat. Commun. **11**, 2747 (2020). <https://doi.org/10.1038/s41467-020-16577-y>

[S55] L. Mei, Z. Cao, T. Ying, R. Yang, H. Peng, G. Wang, L. Zheng, Y. Chen, C.Y. Tang, D. Voiry, H. Wang, A.B. Farimani, Z. Zeng, Simultaneous electrochemical exfoliation and covalent functionalization of MoS_2_ membrane for ion sieving, Adv. Mater. **34**, 2201416 (2022). <https://doi.org/10.1002/adma.202201416>

[S56] P. Kedchaikulrat, I.F.J. Vankelecom, K. Faungnawakij, C. Klaysom, Effects of colloidal TiO_2_ and additives on the interfacial polymerization of thin film nanocomposite membranes, Colloids and Surfaces A **601**, 125046 (2020). <https://doi.org/10.1016/j.colsurfa.2020.125046>

[S57] D. Emadzadeh, W.J. Lau, M. Rahbari-Sisakht, H. Ilbeygi, D. Rana et al., Synthesis, modification and optimization of titanate nanotubes-polyamide thin film nanocomposite (TFN) membrane for forward osmosis (FO) application. Chem. Eng. J. **281**, 243–251 (2015). <https://doi.org/10.1016/j.cej.2015.06.035>

[S58] A.-H M.A. El-Aassar, Improvement of reverse osmosis performance of polyamide thin-film composite membranes using TiO_2_ nanoparticles. Desalin. Water Treat. **55**, 2939–2950 (2015). <https://doi.org/10.1080/19443994.2014.940206>

[S59] A. Al Mayyahi, TiO_2_ polyamide thin film nanocomposite reverses osmosis membrane for water desalination, Membranes **8**, 66 (2018). <https://doi.org/10.3390/membranes8030066>

[S60] S. Jeon, J.H. Lee, Rationally designed in-situ fabrication of thin film nanocomposite membranes with enhanced desalination and anti-biofouling performance, J. Membr. Sci. **615**, 118542 (2020). <https://doi.org/10.1016/j.memsci.2020.118542>

[S61] S. Bian, Y. Wang, F. Xiao, Y. Tong, C. Gao et al., Fabrication of polyamide thin-film nanocomposite reverse osmosis membrane with improved permeability and antibacterial performances using silver immobilized hollow polymer nanospheres. Desalination **539**, 115953 (2022). <https://doi.org/10.1016/j.desal.2022.115953>

[S62] Z. Yang, H. Guo, Z.-k. Yao, Y. Mei, C.Y. Tang, Hydrophilic silver nanoparticles induce selective nanochannels in thin film nanocomposite polyamide membranes, Environ. Sci. Technol. **53**, 5301–5308 (2019). <https://doi.org/10.1021/acs.est.9b00473>

[S63] H. Shen, S. Wang, H. Xu, Y. Zhou, C. Gao, Preparation of polyamide thin film nanocomposite membranes containing silica nanoparticles via an in-situ polymerization of SiCl_4_ in organic solution, J. Membr. Sci. **565**, 145–156 (2018). <https://doi.org/10.1016/j.memsci.2018.08.016>

[S64] M. Zargar, Y. Hartanto, B. Jin, S. Dai, Understanding functionalized silica nanoparticles incorporation in thin film composite membranes: interactions and desalination performance. J. Membr. Sci. **521**, 53–64 (2017). <https://doi.org/10.1016/j.memsci.2016.08.069>

[S65] G.L. Jadav, P.S. Singh, Synthesis of novel silica-polyamide nanocomposite membrane with enhanced properties. J. Membr. Sci. **328**, 257–267 (2009). <https://doi.org/10.1016/j.memsci.2008.12.014>

[S66] H. Huang, Y. Hu, X. Song, J. Kang, D. Chen, Incorporation of dendritic silica nanoparticles for the preparation of high-performance thin-film nanocomposite membranes. J. Appl. Polym. Sci. **141**, e55447 (2024). <https://doi.org/10.1002/app.55447>

[S67] Y. Tong, Y. Wei, H. Zhang, L. Wang, L. Li, F. Xiao, C. Gao, G. Zhu, Fabrication of polyamide thin film nanocomposite membranes with enhanced desalination performance modified by silica nanoparticles formed in-situ polymerization of tetramethoxysilane, J. Environ. Chem. Eng. **11**, 1094115 (2023). <https://doi.org/10.1016/j.jece.2023.109415>

[S68] M. Safarpour, A. Khataee, V. Vatanpour, Thin film nanocomposite reverse osmosis membrane modified by reduced graphene oxide/TiO_2_ with improved desalination performance. J. Membr. Sci. **489**, 43–54 (2015). <https://doi.org/10.1016/j.memsci.2015.04.010>

[S69] M.E.A. Ali, L. Wang, X. Wang, X. Feng, Thin film composite membranes embedded with graphene oxide for water desalination, Desalination **386**, 67–76 (2016). <https://doi.org/10.1016/j.desal.2016.02.034>

[S70] X. Song, Q. Zhou, T. Zhang, H. Xu, Z. Wang, Pressure-assisted preparation of graphene oxide quantum dot-incorporated reverse osmosis membranes: antifouling and chlorine resistance potentials. J. Mater. Chem. A **4**, 16896–16905 (2016). <https://doi.org/10.1039/C6TA06636D>

[S71] S. Li, B. Gao, Y. Wang, B. Jin, Q. Yue et al., Antibacterial thin film nanocomposite reverse osmosis membrane by doping silver phosphate loaded graphene oxide quantum dots in polyamide layer. Desalination **464**, 94–104 (2019). <https://doi.org/10.1016/j.desal.2019.04.029>

[S72] R. Rajakumaran, V. Boddu, M. Kumar, M.S. Shalaby, H. Abdallah et al., Effect of ZnO morphology on GO-ZnO modified polyamide reverse osmosis membranes for desalination. Desalination **467**, 245–256 (2019). <https://doi.org/10.1016/j.desal.2019.06.018>

[S73] G. Hamdy, A. Taher, Enhanced chlorine-resistant and low biofouling reverse osmosis polyimide-graphene oxide thin film nanocomposite membranes for water desalination. Polym. Eng. Sci. **60**, 2567–2580 (2020). <https://doi.org/10.1002/pen.25495>

[S74] B. Wu, N. Zhang, M. Zhang, S. Wang, X. Song et al., Towards a high rejection desalination membrane: the confined growth of polyamide nanofilm induced by alkyl-capped graphene oxide. Membranes **11**, 488 (2021). <https://doi.org/10.3390/membranes11070488>

[S75] A. Inurria, P. Cay-Durgun, D. Rice, H. Zhang, D.-K. Seo et al., Polyamide thin-film nanocomposite membranes with graphene oxide nanosheets: balancing membrane performance and fouling propensity. Desalination **451**, 139–147 (2019). <https://doi.org/10.1016/j.desal.2018.07.004>

[S76] X. Li, S. Chou, R. Wang, L. Shi, W. Fang et al., Nature gives the best solution for desalination: aquaporin-based hollow fiber composite membrane with superior performance. J. Membr. Sci. **494**, 68–77 (2015). <https://doi.org/10.1016/j.memsci.2015.07.040>

[S77] L. Sharma, L. Ye, C. Yong, R. Seetharaman, K. Kho et al., Aquaporin-based membranes made by interfacial polymerization in hollow fibers: visualization and role of aquaporin in water permeability. J. Membr. Sci. **654**, 120551 (2022). <https://doi.org/10.1016/j.memsci.2022.120551>

[S78] Y. Zhao, Y.N. Wang, G.S. Lai, J. Torres, R. Wang, Proteoliposome-incorporated seawater reverse osmosis polyamide membrane: is the aquaporin water channel effect in improving membrane performance overestimated? Environ. Sci. Technol. **56**, 5179–5188 (2022). <https://doi.org/10.1021/acs.est.1c08857>

[S79] X. Li, L. Yang, J. Torres, R. Wang, Engineering ultra-permeable and antifouling water channel-based biomimetic membranes toward sustainable water purification. J. Membr. Sci. Lett. **3**, 100049 (2023). <https://doi.org/10.1016/j.memlet.2023.100049>

[S80] S. Qi, R. Wang, G.K.M. Chaitra, J. Torres, X. Hu et al., Aquaporin-based biomimetic reverse osmosis membranes: stability and long term performance. J. Membr. Sci. **508**, 94–103 (2016). <https://doi.org/10.1016/j.memsci.2016.02.013>

[S81] Y. Li, S. Qi, M. Tian, W. Widjajanti, R. Wang, Fabrication of aquaporin-based biomimetic membrane for seawater desalination. Desalination **467**, 103–112 (2019). <https://doi.org/10.1016/j.desal.2019.06.005>

[S82] Y. He, H. Hoi, S. Abraham, C.D. Montemagno, Highly permeable biomimetic reverse osmosis membrane with amphiphilic peptide stabilized aquaporin as water filtering agent. J. Appl. Polym. Sci. **135**, e46169 (2018). <https://doi.org/10.1002/app.46169>

[S83] M. Di Vincenzo, A. Tiraferri, V.E. Musteata, S. Chisca, R. Sougrat, L.B. Huang, S.P. Nunes, M. Barboiu, Biomimetic artificial water channel membranes for enhanced desalination, Nat. Nanotechnol. **16**, 190–196 (2021). <https://doi.org/10.1038/s41565-020-00796-x>

[S84] M. Di Vincenzo, A. Tiraferri, V.E. Musteata, S. Chisca, M. Deleanu, F. Ricceri, D. Cot, S.P. Nunes, M. Barboiu, Tunable membranes incorporating artificial water channels for high-performance brackish/low-salinity water reverse osmosis desalination, Proc. Natl. Acad. Sci. U.S.A. **118**, e2022200118 (2021). <https://doi.org/10.1073/pnas.2022200118>

[S85] Q. Wang, J. Sun, W. Xue, G. Zhao, W. Ding et al., Effect of carbon nanotube nanochannel on the separation performance of thin-film nanocomposite (TFN) membranes. Desalination **546**, 116216 (2023). <https://doi.org/10.1016/j.desal.2022.116216>

[S86] J. Sun, W. Liu, W. Ding, G. Zhao, Q. Zhang et al., Improved water flux and separation of polyamide reverse osmosis membranes by trace loading of biomimetic modified carbon nanotubes. ACS Appl. Nano Mater. **7**, 17840–17854 (2024). <https://doi.org/10.1021/acsanm.4c03002>

[S87] A. Güvensoy-Morkoyun, S. Velioğlu, M.G. Ahunbay, Ş.B. Tantekin-Ersolmaz, Desalination potential of aquaporin-inspired functionalization of carbon nanotubes: bridging between simulation and experiment. ACS Appl. Mater. Interfaces **14**, 28174–28185 (2022). <https://doi.org/10.1021/acsami.2c03700>

[S88] H.D. Lee, H.W. Kim, Y.H. Cho, H.B. Park, Experimental evidence of rapid water transport through carbon nanotubes embedded in polymeric desalination membranes, Small **10**, 2653–2660 (2014). <https://doi.org/10.1002/smll.201303945>

[S89] M. Zhao, S. Fu, H. Zhang, H. Huang, Y. Wei et al., Enhanced separation and antifouling performance of reverse osmosis membrane incorporated with carbon nanotubes functionalized by atom transfer radical polymerization. RSC Adv. **7**, 46969–46979 (2017). <https://doi.org/10.1039/C7RA08351C>

[S90] J. Farahbakhsh, M. Delnavaz, V. Vatanpour, Investigation of raw and oxidized multiwalled carbon nanotubes in fabrication of reverse osmosis polyamide membranes for improvement in desalination and antifouling properties. Desalination **410**, 1–9 (2017). <https://doi.org/10.1016/j.desal.2017.01.031>

[S91] Y. Baek, H.J. Kim, S.-H. Kim, J.-C. Lee, J. Yoon, Evaluation of carbon nanotube-polyamide thin-film nanocomposite reverse osmosis membrane: surface properties, performance characteristics and fouling behavior. J. Ind. Eng. Chem. **56**, 327–334 (2017). <https://doi.org/10.1016/j.jiec.2017.07.028>

[S92] M.L. Lind, A.K. Ghosh, A. Jawor, X. Huang, W. Hou et al., Influence of zeolite crystal size on zeolite-polyamide thin film nanocomposite membranes. Langmuir **25**, 10139–10145 (2009). <https://doi.org/10.1021/la900938x>

[S93] M.L. Lind, D. Eumine Suk, T.V. Nguyen, E.M. Hoek, Tailoring the structure of thin film nanocomposite membranes to achieve seawater RO membrane performance. Environ. Sci. Technol. **44**, 8230–8235 (2010). <https://doi.org/10.1021/es101569p>

[S94] M. Fathizadeh, A. Aroujalian, A. Raisi, Effect of added NaX nano-zeolite into polyamide as a top thin layer of membrane on water flux and salt rejection in a reverse osmosis process. J. Membr. Sci. **375**, 88–95 (2011). <https://doi.org/10.1016/j.memsci.2011.03.017>

[S95] H. Dong, X.-Y. Qu, L. Zhang, L.-H. Cheng, H.-L. Chen et al., Preparation and characterization of surface-modified zeolite-polyamide thin film nanocomposite membranes for desalination. Desalin. Water Treat. **34**, 6–12 (2011). <https://doi.org/10.5004/dwt.2011.2789>

[S96] N. Ma, J. Wei, R. Liao, C.Y. Tang, Zeolite-polyamide thin film nanocomposite membranes: towards enhanced performance for forward osmosis. J. Membr. Sci. **405**, 149–157 (2012). <https://doi.org/10.1016/j.memsci.2012.03.002>

[S97] H. Huang, X. Qu, X. Ji, X. Gao, L. Zhang et al., Acid and multivalent ion resistance of thin film nanocomposite RO membranes loaded with silicalite-1 nanozeolites. J. Mater. Chem. A **1**, 11343–11349 (2013). <https://doi.org/10.1039/C3TA12199B>

[S98] H. Huang, X. Qu, H. Dong, L. Zhang, H. Chen, Role of NaA zeolites in the interfacial polymerization process towards a polyamide nanocomposite reverse osmosis membrane. RSC Adv. **3**, 8203–8207 (2013). <https://doi.org/10.1039/C3RA40960K>

[S99] M.M. Pendergast, A.K. Ghosh, E.M.V. Hoek, Separation performance and interfacial properties of nanocomposite reverse osmosis membranes. Desalination **308**, 180–185 (2013). <https://doi.org/10.1016/j.desal.2011.05.005>

[S100] H. Dong, L. Zhao, L. Zhang, H. Chen, C. Gao et al., High-flux reverse osmosis membranes incorporated with NaY zeolite nanoparticles for brackish water desalination. J. Membr. Sci. **476**, 373–383 (2015). <https://doi.org/10.1016/j.memsci.2014.11.054>

[S101] H. Marioryad, A.M. Ghaedi, D. Emadzadeh, M.M. Baneshi, A. Vafaei et al., A thin film nanocomposite reverse osmosis membrane incorporated with S-beta zeolite nanoparticles for water desalination. ChemistrySelect **5**, 1972–1975 (2020). <https://doi.org/10.1002/slct.201904084>

[S102] J. Duan, Y. Pan, F. Pacheco, E. Litwiller, Z. Lai et al., High-performance polyamide thin-film-nanocomposite reverse osmosis membranes containing hydrophobic zeolitic imidazolate framework-8. J. Membr. Sci. **476**, 303–310 (2015). <https://doi.org/10.1016/j.memsci.2014.11.038>

[S103] M. Kadhom, W. Hu, B. Deng, Thin film nanocomposite membrane filled with metal-organic frameworks UiO-66 and MIL-125 nanoparticles for water desalination. Membranes **7**, 31 (2017). <https://doi.org/10.3390/membranes7020031>

[S104] D. Ma, S.B. Peh, G. Han, S.B. Chen, Thin-film nanocomposite (TFN) membranes incorporated with super-hydrophilic metal-organic framework (MOF) UiO-66: toward enhancement of water flux and salt rejection. ACS Appl. Mater. Interfaces **9**, 7523–7534 (2017). <https://doi.org/10.1021/acsami.6b14223>

[S105] L. Liu, X. Xie, S. Qi, R. Li, X. Zhang et al., Thin film nanocomposite reverse osmosis membrane incorporated with UiO-66 nanoparticles for enhanced boron removal. J. Membr. Sci. **580**, 101–109 (2019). <https://doi.org/10.1016/j.memsci.2019.02.072>

[S106] Y. Wen, Y. Chen, Z. Wu, M. Liu, Z. Wang, Thin-film nanocomposite membranes incorporated with water stable metal-organic framework CuBTTri for mitigating biofouling. J. Membr. Sci. **582**, 289–297 (2019). <https://doi.org/10.1016/j.memsci.2019.04.016>

[S107] Z. Zhai, N. Zhao, W. Dong, P. Li, H. Sun et al., *In situ* assembly of a zeolite imidazolate framework hybrid thin-film nanocomposite membrane with enhanced desalination performance induced by noria-polyethyleneimine codeposition. ACS Appl. Mater. Interfaces **11**, 12871–12879 (2019). <https://doi.org/10.1021/acsami.9b01237>

[S108] Y. Liu, X.-P. Wang, Z.-A. Zong, R. Lin, X.-Y. Zhang et al., Thin film nanocomposite membrane incorporated with 2D-MOF nanosheets for highly efficient reverse osmosis desalination. J. Membr. Sci. **653**, 120520 (2022). <https://doi.org/10.1016/j.memsci.2022.120520>

[S109] P. Hu, B. Yuan, Q. Jason Niu, N. Wang, S. Zhao et al., *In situ* assembled zeolite imidazolate framework nanocrystals hybrid thin film nanocomposite membranes for brackish water desalination. Sep. Purif. Technol. **293**, 121134 (2022). <https://doi.org/10.1016/j.seppur.2022.121134>

[S110] R.R. Gonzales, M.J. Park, T.-H. Bae, Y. Yang, A. Abdel-Wahab et al., Melamine-based covalent organic framework-incorporated thin film nanocomposite membrane for enhanced osmotic power generation. Desalination **459**, 10–19 (2019). <https://doi.org/10.1016/j.desal.2019.02.013>

[S111] G. Yang, Z. Zhang, C. Yin, X. Shi, Y. Wang, Polyamide membranes enabled by covalent organic framework nanofibers for efficient reverse osmosis. J. Polym. Sci. **60**, 2999–3008 (2022). <https://doi.org/10.1002/pol.20210664>

[S112] L. Xu, B. Shan, C. Gao, J. Xu, Multifunctional thin-film nanocomposite membranes comprising covalent organic nanosheets with high crystallinity for efficient reverse osmosis desalination, J. Membr. Sci. **593**, 117398 (2020). <https://doi.org/10.1016/j.memsci.2019.117398>

[S113] X. Wang, Q. Li, J. Zhang, H. Huang, S. Wu, Y. Yang, Novel thin-film reverse osmosis membrane with MXene Ti_3_C_2_T_x_ embedded in polyamide to enhance the water flux, anti-fouling and chlorine resistance for water desalination, J. Membr. Sci. **603**, 118036 (2020). <https://doi.org/10.1016/j.memsci.2020.118036>

[S114] K. Zarshenas, H. Dou, S. Habibpour, A. Yu, Z. Chen, Thin film polyamide nanocomposite membrane decorated by polyphenol-assisted Ti_3_C_2_T*_x_* MXene nanosheets for reverse osmosis. ACS Appl. Mater. Interfaces **14**, 1838–1849 (2022). <https://doi.org/10.1021/acsami.1c16229>

[S115] Y. Li, S. Yang, K. Zhang, B. Van der Bruggen, Thin film nanocomposite reverse osmosis membrane modified by two dimensional laminar MoS_2_ with improved desalination performance and fouling-resistant characteristics. Desalination **454**, 48–58 (2019). <https://doi.org/10.1016/j.desal.2018.12.016>

[S116] A. Güvensoy-Morkoyun, S. Kürklü-Kocaoğlu, C. Yıldırım, S. Velioğlu, H.E. Karahan et al., Carbon nanotubes integrated into polyamide membranes by support pre-infiltration improve the desalination performance. Carbon **185**, 546–557 (2021). <https://doi.org/10.1016/j.carbon.2021.09.021>

[S117] J.H. Lee, H.S. Kim, E.T. Yun, S.Y. Ham, J.H. Park, C.H. Ahn, S.H. Lee, H.D. Park, Vertically aligned carbon nanotube membranes: water purification and beyond, Membranes (Basel) **10**, 273 (2020). <https://doi.org/10.3390/membranes10100273>

[S118] M.J. Borgnia, D. Kozono, G. Calamita, P.C. Maloney, P. Agre, Functional reconstitution and characterization of AqpZ, the E. coli water channel protein, J. Mol. Biol. **291**, 1169–1179 (1999). <https://doi.org/10.1006/jmbi.1999.3032>

[S119] X. Li, R. Wang, C. Tang, A. Vararattanavech, Y. Zhao, J. Torres, T. Fane, Preparation of supported lipid membranes for aquaporin Z incorporation, Colloids Surf. B **94**, 333–340 (2012). <https://doi.org/10.1016/j.colsurfb.2012.02.013>

[S120] A. Fuwad, H. Ryu, E.D. Han, J.-H. Lee, N. Malmstadt, Y.-R. Kim, Y.H. Seo, S.M. Kim, T.-J. Jeon, Highly permeable and shelf-stable aquaporin biomimetic membrane based on an anodic aluminum oxide substrate, npj Clean Water **7**, 11 (2024). <https://doi.org/10.1038/s41545-024-00301-0>

[S121] M.A. Tofighy, Y. Shirazi, T. Mohammadi, A. Pak, Salty water desalination using carbon nanotubes membrane. Chem. Eng. J. **168**, 1064–1072 (2011). <https://doi.org/10.1016/j.cej.2011.01.086>

[S122] H. Azami, M.R. Omidkhah, Vertically aligned carbon nanotube membrane: synthesis, characterization and application in salt water desalination, Advances in Environmental Technology **6**, 173–189 (2020). <https://doi.org/10.22104/AET.2020.4707.1281>.

[S123] M.C. Duke, J. O’Brien-Abraham, N. Milne, B. Zhu, J.Y.S. Lin, J.C. Diniz da Costa, Seawater desalination performance of MFI type membranes made by secondary growth, Sep. Purif. Technol. **68**, 343–350 (2009). <https://doi.org/10.1016/j.seppur.2009.06.003>

[S124] G.A. Ozin, A. Kuperman, A. Stein, Advanced zeolite, materials science. Angew. Chem. Int. Ed. **28**, 359–376 (1989). <https://doi.org/10.1002/anie.198903591>

[S125] X. Wang, Q. Lyu, T. Tong, K. Sun, L.C. Lin, C.Y. Tang, F. Yang, M.D. Guiver, X. Quan, Y. Dong, Robust ultrathin nanoporous MOF membrane with intra-crystalline defects for fast water transport, Nat. Commun. **13**, 1–11 (2022). <https://doi.org/10.1038/s41467-021-27873-6>

[S126] I. Gadwal, G. Sheng, R.L. Thankamony, Y. Liu, H. Li et al., Synthesis of sub-10 nm two-dimensional covalent organic thin film with sharp molecular sieving nanofiltration. ACS Appl. Mater. Interfaces **10**, 12295–12299 (2018). <https://doi.org/10.1021/acsami.7b19450>

[S127] H. Yang, X. Cheng, X. Cheng, F. Pan, H. Wu et al., Highly water-selective membranes based on hollow covalent organic frameworks with fast transport pathways. J. Membr. Sci. **565**, 331–341 (2018). <https://doi.org/10.1016/j.memsci.2018.08.043>

[S128] F. Pan, W. Guo, Y. Su, N.A. Khan, H. Yang, Z. Jiang, Direct growth of covalent organic framework nanofiltration membranes on modified porous substrates for dyes separation, Sep. Purif. Technol. **215**, 582–589 (2019). <https://doi.org/10.1016/j.seppur.2019.01.064>

[S129] H. Huang, Y. Mao, Y. Ying, Y. Liu, L. Sun, X. Peng, Salt concentration, pH and pressure controlled separation of small molecules through lamellar graphene oxide membranes, Chem. Commun. **49**, 5963–5965 (2013). <https://doi.org/10.1039/c3cc41953c>

[S130] J. Wang, X. Gao, H. Yu, Q. Wang, Z. Ma et al., Accessing of graphene oxide (GO) nanofiltration membranes for microbial and fouling resistance. Sep. Purif. Technol. **215**, 91–101 (2019). <https://doi.org/10.1016/j.seppur.2019.01.018>

[S131] J. Wang, Z. Zhang, J. Zhu, M. Tian, S. Zheng, F. Wang, X. Wang, L. Wang, Ion sieving by a two-dimensional Ti_3_C_2_T_x_ alginate lamellar membrane with stable interlayer spacing, Nat. Commun. **11**, 3540 (2020). <https://doi.org/10.1038/s41467-020-17373-4>

[S132] L. Ding, L. Li, Y. Liu, Y. Wu, Z. Lu, J. Deng, Y. Wei, J. Caro, H. Wang, Effective ion sieving with Ti_3_C_2_T_x_ MXene membranes for production of drinking water from seawater, Nat. Sustain. **3**, 296–302 (2020). <https://doi.org/10.1038/s41893-020-0474-0>

[S133] Y. Zhang, D. Chen, N. Li, Q. Xu, H. Li, J. He, J. Lu, High-performance and stable two-dimensional MXene-polyethyleneimine composite lamellar membranes for molecular separation, ACS Appl. Mater. Interfaces **14**, 10237–10245 (2022). <https://doi.org/10.1021/acsami.1c20540>

[S134] L. Ding, L. Li, Y. Liu, Y. Wu, Z. Lu, J. Deng, Y. Wei, J. Caro, H. Wang, Effective ion sieving with Ti_3_C_2_T_x_ MXene membranes for production of drinking water from seawater, Nat. Sustain. **3**, 296–302 (2020). <https://doi.org/10.1038/s41893-020-0474-0>

[S135] T. Habib, X. Zhao, S.A. Shah, Y. Chen, W. Sun, H. An, J.L. Lutkenhaus, M. Radovic, M.J. Green, Oxidation stability of Ti_3_C_2_T_x_ MXene nanosheets in solvents and composite films, NPJ 2D Mater. Appl. **3**, 8 (2019). <https://doi.org/10.1038/s41699-019-0089-3>

[S136] Q. Lin, Y. Liu, G. Zeng, X. Li, B. Wang et al., Bionics inspired modified two-dimensional MXene composite membrane for high-throughput dye separation. J. Environ. Chem. Eng. **9**, 105711 (2021). <https://doi.org/10.1016/j.jece.2021.105711>

[S137] J. Kaur, M. Singh, C. Dell'Aversana, R. Benedetti, P. Giardina, M. Rossi, M. Valadan, A. Vergara, A. Cutarelli, A.M.I. Montone, L. Altucci, F. Corrado, A. Nebbioso, C. Altucci, Biological interactions of biocompatible and water-dispersed MoS_2_ nanosheets with bacteria and human cells, Sci. Rep. **8,** 16386 (2018). <https://doi.org/10.1038/s41598-018-34679-y>
